# Discovery of Two New Sorbicillinoids by Overexpression of the Global Regulator LaeA in a Marine-Derived Fungus *Penicillium dipodomyis* YJ-11

**DOI:** 10.3390/md17080446

**Published:** 2019-07-28

**Authors:** Jing Yu, Huan Han, Xianyan Zhang, Chuanteng Ma, Chunxiao Sun, Qian Che, Qianqun Gu, Tianjiao Zhu, Guojian Zhang, Dehai Li

**Affiliations:** 1 Key Laboratory of Marine Drugs, Chinese Ministry of Education, School of Medicine and Pharmacy, Ocean University of China, Qingdao 266003, China; 2 Laboratory for Marine Drugs and Bioproducts, Pilot National Laboratory for Marine Science and Technology (Qingdao), Qingdao 266237, China

**Keywords:** genome mining, global regulator, LaeA, overexpression, *Penicillium dipodomyis*, sorbicillinoids

## Abstract

Overexpression of the global regulator LaeA in a marine-derived fungal strain of *Penicillium dipodomyis* YJ-11 induced obvious morphological changes and metabolic variations. Further chemical investigation of the mutant strain afforded a series of sorbicillinoids including two new ones named 10,11-dihydrobislongiquinolide (**1**) and 10,11,16,17-tetrahydrobislongiquinolide (**2**), as well as four known analogues, bislongiquinolide (**3**), 16,17-dihydrobislongiquinolide (**4**), sohirnone A (**5**), and 2′,3′-dihydrosorbicillin (**6**). The results support that the global regulator LaeA is a useful tool in activating silent gene clusters in *Penicillium* strains to obtain previously undiscovered compounds.

## 1. Introduction

Filamentous fungi have proven to be important sources of bioactive natural products for development of new drug leads. As the traditional approach (cultivation of microorganisms, chemical extraction of the produced metabolites, and final structure and bioactivity elucidations) has been continuously applied in discovery of new secondary metabolites, it has become a frequent issue that known structures are repeatedly discovered, while a big portion of biosynthetic genes are not expressed under current culturing technologies also termed as “silent” or “cryptic” genes. To increase the silent metabolic potential of the microbial producers, a variety of approaches such as heterologous expression, epigenetic regulation, transcriptional regulation and ribosome engineering, have been developed to affect the biosynthetic process in different gene regulation levels [1,2,3], among which, transcriptional factor regulation is often adopted because it is feasible and effective, and from which unexpected new secondary metabolites could be obtained by activation of the silent genes.

LaeA is an effective global regulator which was first discovered from *Aspergillus nidulans* and *A. fumigatus* by Jin Woo Bok and Nancy P. Keller in 2004 [4], and proved to be able to influence fungi in many aspects, such as increasing [5,6,7] or reducing secondary metabolite production [8], activating cryptic gene clusters [9], asexual and sexual differentiation as well as changes in phenotype including sporulation [5,9] and pigmentations [5]. Later on, LaeA gene analogues with similar functions were reported from other fungal species such as *Penicillium chrysogenum* [10], *Monascus ruber* [11], *Alternaria alternate* [12], and *Dothistroma septosporum* [8].

**Figure 1:**
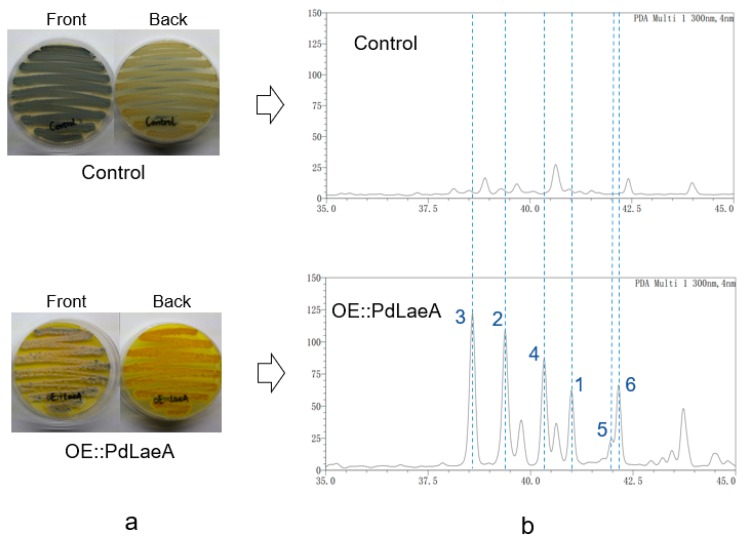
(**a**) Morphologies of the control strain and OE::PdLaeA strain of *P. dipodomyis* YJ- 11 after incubating at 28 °C for 5 days for sporulation. (**b**) HPLC analysis of the extracts from the control strain and OE::PdLaeA strain of *P. dipodomyis* YJ-11.

As a part of our ongoing work searching for diverse secondary metabolites from marine derived fungi, we recently isolated one filamentous fungi *Penicillium dipodomyis* YJ-11 from a marine sediment sample collected in Jiaozhou Bay of Qingdao. In order to activate the silent metabolic potential and obtain diversified secondary metabolites, we overexpressed the native global regulator of PdLaeA in *P. dipodomyis* YJ-11 and the mutant showed changes both in morphologies (sporulation and pigmentations) and metabolic profiles in contrast to the control (Figure 1). Further chemical studies on the mutant strain led to the isolation of two new compounds, 10,11-dihydrobislongiquinolide (**1**) and 10,11,16,17-tetrahydrobislongiquinolide (**2**), together with four known analogues, bislongiquinolide (**3**), also named as bisorbibutenolide, 16,17-dihydrobislongiquinolide (**4**), also named as dihydrotrichotetronine, sohirnone A (**5**), and 2′,3′-dihydrosorbicillin (**6**). 

## 2. Results and Discussion

The LaeA gene analogue PdLaeA was identified via Localblast, using a LaeA gene (*Aspergillus nidulans*, Q6TLK5.1) as a query. The PdLaeA gene was then cloned from genomic DNA of the strain *P. dipodomyis* YJ-11. The total size of the gene is 1283 bp and the predicted open reading frame (ORF) is 1053 bp, which may encode a polypeptide of 350 amino acids. BLAST analysis by NCBI indicated that the PdLaeA protein had 95% sequence identity to the protein of PcLaeA (*P. citrinum*, BAL61197.1), PrLaeA (*P. roqueforti FM164*, CDM34701.1), PdiLaeA (*P. digitatum PHI26*, EKV10385.1), and PdiLaeA (*P. digitatum Pd1*, XP_014530787.1). Phylogenetic analysis revealed that PdLaeA is mostly related to PcLaeA (Figure S2 in Supplementary Materials). Sequence analysis via InterProScan showed that the PdLaeA protein was an S-adenosyl-l-methionine-dependent methyltransferase (IPR029063), which is consistent with the putative mechanism of LaeA genes [13,14,15]. 

The PdLaeA in *P. dipodomyis* YJ-11 was amplified by specific primers (Table S1) and ligated into the vector pZeo using restriction sites XhoI and XbaI. The recombinant vector was transformed to *P. dipodomyis* YJ-11 to generate the OE::PdLaeA mutants (the mutant of *P. dipodomyis* YJ-11 harboring vacant pZeo was also generated as a control). The mutants showed obvious changes on both spore morphology and pigment formation. Followed by fermentation in PDB media with shaking at 28 °C for 9 days, high performance liquid chromatography (HPLC) analysis also showed a series of new peaks presenting in the extract of the OE::PdLaeA mutant compared with that of the control strain (Figure 1), indicating changes in secondary metabolite production.

**Figure 2:**
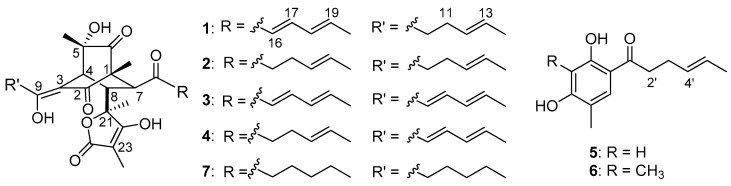
Structures of compounds **1**–**6** isolated from the strain OE::PdLaeA P*enicillium dipodomyis* YJ-11 and hydrogenation product **7**.

For exploring the structures for the activation products, the OE::PdLaeA mutant was cultured in larger scale (10 L). Guided by UPLC-MS data, the EtOAc extract (12 g) of the fermentation was fractionated by octadecyl silane chemically bonded silica (ODS) medium performance liquid chromatography (MPLC) and then HPLC to yield compounds 10,11-dihydrobislongiquinolide (**1**, 11 mg), 10,11,16,17-tetrahydrobislongiquinolide (**2**, 35 mg), bislongiquinolide which also had been named as bisorbibutenolide [16,17] (**3**, 18 mg), 16,17-dihydrobislongiquinolide (also named as dihydrotrichotetronine [18,19], **4**, 84 mg), sohirnone A (**5**, 6 mg), and 2′,3′-dihydrosorbicillin (**6**, 5 mg) (Figure 2). The known compounds **3**–**6** were obtained and identified by the comparation of ^1^H NMR data and mass spectroscopy with the literature refs. [16,17,18,19,20,21,22]. Compounds **3**, **5,** and **6** which had been isolated in our laboratory before [20], were also confirmed by HPLC analysis using standard samples.

**Figure 3:**
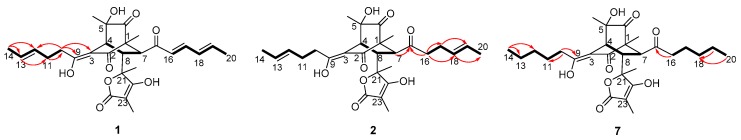
COSY and key HMBC correlations of compounds **1**,**2** and **7**.

Compound **1** was obtained as a yellow powder with the molecular formula C_28_H_34_O_8_ supported by the high resolution electrospray ionization mass spectroscopy (HRESIMS) peak at *m*/*z* 497.2185 [M − H]^−^ (calcd. 497.2170). The ^1^H NMR spectrum of **1** revealed six methyl proton signals, two methylene proton signals, nine methine signals including six olefinic ones at 5.0–7.5 ppm, and three aliphatic ones at 2.8–3.5 ppm. In the ^13^C NMR spectrum, in addition to the six methyls, two methylenes and nine methines which were assignable carbon signals, there were 11 quaternary carbons. The unsaturation degree further suggested the presence of three rings in the structure. The ^1^H and ^13^C NMR data (Table 1) were very similar to those of bislongiquinolide (**3**) [16,17] indicating that they had the same core structure. The major differences were the replacement of one double bond (in **3**) by a single bond (in **1**). Careful comparison of the ^13^C NMR data of C_9_-C_14_ (*δ*_C_ 181.3, 32.5, 28.4, 129.1, 126.9, 17.8 respectively) with those of compound **3** indicated the *Δ*^10^ double bond was reduced. The key homonuclear chemical shift correlation spectroscopy (COSY) correlations of H-10/H-11/H-12/H-13/H-14 together with ^1^H detected heteronuclear multiple bond correlation (HMBC) correlations from H-11 (*δ*_H_ 2.42) to C-10 (*δ*_C_ 32.5), from H-10 (*δ*_H_ 2.51) to C-12 (*δ*_C_ 129.1), C-9 (*δ*_C_ 181.3), and C-3 (*δ*_C_ 108.4) (Figure 3) confirmed the structure of **1** as the 10,11-hydrogeneted analogue of **3**, which was named as 10,11-dihydrobislongiquinolide. 

Compound **2** was obtained as a yellow oil and it was analyzed by HRESIMS (*m*/*z* 499.2343 [M − H]^−^, calcd. 499.2326) for the molecular formula C_28_H_36_O_8_, which was one double bond equivalent (DBE) less than **1**. The ^1^H NMR spectrum of **2** revealed six methyl proton signals, four methylene proton signals, and seven methine protons with four olefinic ones (*δ*_H_ 5.0–6.0) and three aliphatic ones (*δ*_H_ 2.6–3.5). The ^1^H and ^13^C NMR data (Table 1) were very similar to those of **1** except that one double bond was hydrogenated in **2**. Careful comparison of the ^13^C-NMR data of C_15_-C_20_ with those of compound **1** indicated that the C_16_-C_17_ double bond was reduced, which was further confirmed by the COSY correlations (H-16/H-17/H-18, H-19/H-20) and HMBC correlations from H-18 (*δ*_H_ 5.34) to C-20 (*δ*_C_ 17.8), from H-17 (*δ*_H_ 2.11 and *δ*_H_ 2.19) to C-19 (*δ*_C_ 126.8), from H-16 (*δ*_H_ 2.45–2.60) to C-15 (*δ*_C_ 213.2), C-17 (*δ*_C_ 25.9), and C-18 (*δ*_C_ 128.5) (Figure 3). Thus, the structure of **2** was established and named as 10,11,16,17-tetrahydrobislongiquinolide. 

**Figure 4:**
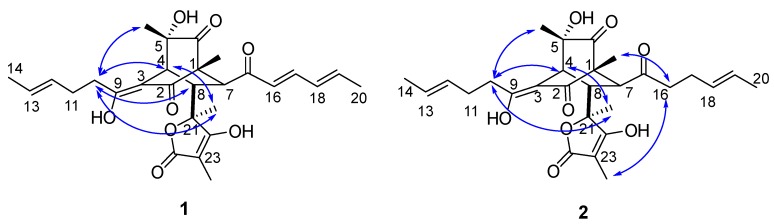
Key NOESY correlations of compounds **1** and **2**.

**Figure 5:**
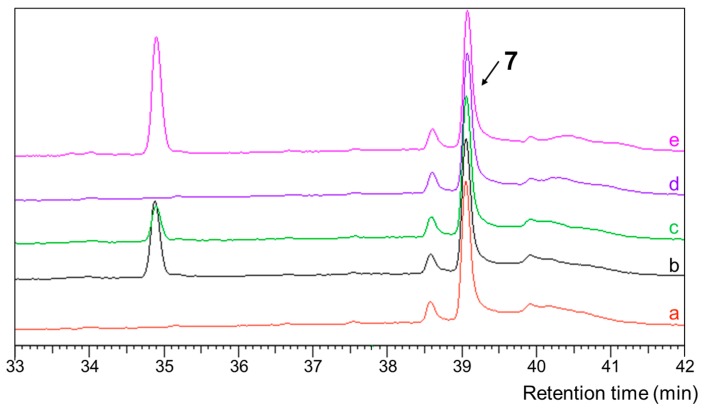
HPLC analysis of hydrogenation reaction products: (**a–d**) represent the reaction products of compounds **1–4** respectively and (**e**) is a co-injection of all the reaction products of compounds **1–4.**

**Figure 6:**
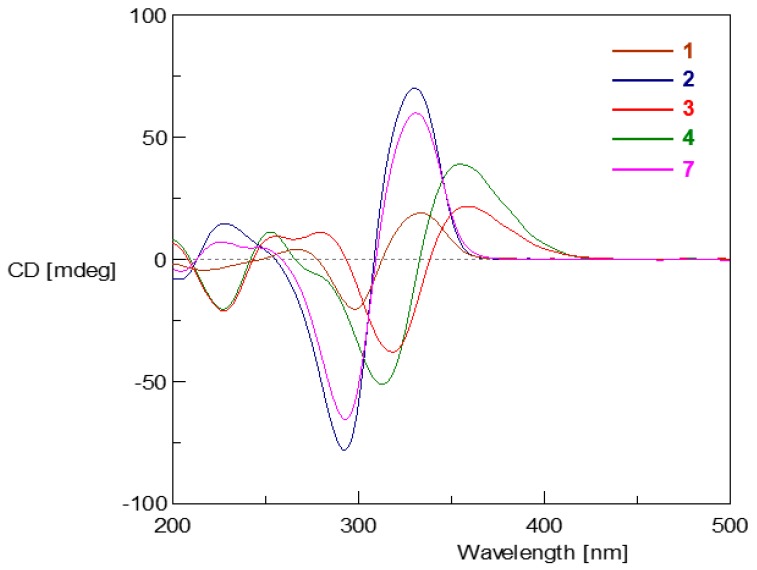
Circular dichroism (CD) spectra of **1**–**4** and **7**.

The relative and absolute configurations of compounds **1** and **2** were determined by nuclear overhauser effect spectroscopy (NOESY) correlations (Figure 4), coupling constants (Table 1), hydrogenation reaction (Figure 5), circular dichroism (CD) spectra (Figure 6), and biogenetic considerations. In compound **1**, the *E* geometries of the double bonds in the side chains were deduced by the large coupling constants (15.0 Hz). In compound **2**, the NOESY correlation (Figure 4) between 1-CH_3_ (*δ*_H_ 1.13) and H-16 (*δ*_H_ 2.45–2.60) suggested the same orientation of 1-CH_3_ and the chain from C-7 through C-20; the NOESY correlation between H-4 (*δ*_H_ 3.42) and H- 10 (*δ*_H_ 2.45–2.60) indicated a *Z* geometry of *Δ*^3, 9^ and located H-4 to the same side of the chain from C-9 through C-14, which was supported by the similar chemical shifts as reported for the known structure of bislongiquinolide [17] and bisorbibutenolide [16]. The NOESY correlation of 5-CH_3_ (*δ*_H_ 1.31)/H-10 suggested that 5-CH_3_ faced to C-3 in the bicyclo[2.2.2] ring. The 7R*, 8S* relative configuration was suggested by the coupling constant (^3^*J*_H-7, H-8_ = 6.6 Hz) [16]. The NOESY correlation of H-16/23-CH_3_ (*δ*_H_ 1.71) indicated that the 23-CH_3_ pointed to the direction of the side chain from C- 15 to C-20, while the NOESY correlations of H-10/21-CH_3_ and H-4/21-CH_3_ oriented 21-CH_3_ to another side chain from C-9 to C-14 and H-4. Thus, the relative configuration of compound **2** should be 1R*, 4S*, 5S*, 7R*, 8S*, 21S*, which adopted the same configuration of the bicyclo[2.2.2]octanedione core as the reported analogues [16,17].

To confirm the consistent configurations of the co-isolated compounds **1**–**4**, hydrogenation over palladium on carbon was performed respectively. HPLC analysis of the reaction products showed that compounds **1**–**4** gave the same hydrogenation product (Figure 5), which suggested identical configurations of compounds **1**–**4** core structures. The hydrogenated product, named octahydrobislongiquinolide (**7**), was isolated by HPLC. 

Compound **7** was obtained as a white powder with the molecular formula C_28_H_40_O_8_ supported by the HRESIMS peak at *m*/*z* 527.2608 [M + Na]^+^ (calcd. 527.2615). In addition to the signals expected for the bicyclo[2.2.2] core structure and the butyrolactone ring, the 1D NMR spectra of **7** shows signals for two methyls, eight methylenes, and no olefinic proton indicating that the double bonds in the sorbyl side chains were hydrogenated thoroughly. The key COSY correlations of H-10/H-11/H-12/H-13/H-14 together with HMBC correlations from H-14 (*δ*_H_ 0.87) to C-13 (*δ*_C_ 31.9) and C-12 (*δ*_C_ 22.5), from H-10 (*δ*_H_ 2.40–2.50) to C-11 (*δ*_C_ 25.2) and C-9 (*δ*_C_ 182.4) (Figure 3) confirmed the structure of one sidechain. Similarly, the key COSY correlations of H-16/H-17/H-18/H-19/H-20 together with HMBC correlations from H-16 (*δ*_H_ 2.40–2.50) to C-15 (*δ*_C_ 214.3), from H-19 (*δ*_H_ 1.20–1.30) to C-18 (*δ*_C_ 31.0), from H-20 (*δ*_H_ 0.89) to C-18 (*δ*_C_ 31.0) (Figure 3) confirm the structure of the other sidechain. Thus, the structure of **7** was established and named as octahydrobislongiquinolide. 

Therefore, the absolute configurations of **1** and **2** were proposed to be the same as the co-isolated **3** and **4**, which were also supported by the optical rotation values (**1**, **2**, **3**, **4**: +35.6, +71.8, +105 [17], +350 [19]), the biogenetic consideration, and the similar trend in CD curves (Figure 6). Moreover, in the CD spectrum as shown in Figure 6, the curves of compounds **1**–**4** and **7** clustered into two groups (**1**, **2,** and **7** were in one group and **3** and **4** belonged to the other), which suggested that the double bond at C-10 on the 3-sorbyl substitution made a dominant contribution to the shape of the CD spectrum compared with the double bonds at other positions such as C-12, C-16 and C-18 in bicyclo[2.2.2]octanedione containing sorbicillinoid structures.

The cytotoxicities of compounds **1**–**4** were evaluated using HL-60, K562, BEL-7402, HCT-116, A549, Hela, L-02, MGC-803, SH-SY5Y, PC-3, H446, U87, MDA-MB-231, HO8910, ASPC-1 and MCF- 7 cell lines, but none of them presented a cytotoxic effect at 30 µM. The antimicrobial activity was also evaluated, with no activity detected under the concentration of 30 *µ*M either. Compounds **1**–**4** exhibited identical weak siderophore activity with chrome azurol sulfonate (CAS) with an ED_50_ value of 400 µM (deferoxamine mesylate was used as a positive control with an ED_50_ value of 100 µM). Inspired by the radical scavenging activity of the known compound **3** [16], 1,1-diphenyl-2-picrylhydrazyl radical 2,2-diphenyl-1-(2,4,6-trinitrophenyl)hydrazyl (DPPH) radical scavenging assay was used to test the radical scavenging activity of compounds **2**–**4** (a paucity of compound **1** prevented analysis), and they showed similar effects with ED_50_ values of 166.7 μM, 183.3 μM, and 89.6 μM (the value of ascorbic acid was 27.3 μM as positive control). 

The sorbicillinoids are a family of hexaketide metabolites, with the presence of the sorbyl sidechain as a unique structural feature. Monomeric, dimeric, trimeric, and nitrogen-containing metabolites constitute the sorbicillinoids, among which dimers are the most common, with potential bioactivities as drug leads [23,24]. The compounds **1**–**4** in this report are an expansion of the library of the bridged bicyclic bisorbicillinoids with the bicyclo[2.2.2]octanedione core structure. In contrast to the previous discovery strategies of conventional extraction and separation, biosynthesis or total synthesis, it is the first report of sorbicillinoid discovery by activating silent gene clusters. The above result shows that overexpression of the global regulator LaeA is a useful method to discover new natural products by activating silent biosynthetic gene clusters. 

## 3. Materials and Methods

### 3.1. General Experimental Procedures

DNA restriction enzymes were used as recommended by the manufacturer (New England Biolabs, NEB, Beijing, China). Polymerase chain reaction (PCR) was performed using *TransStart^®^ Fastpfu* DNA Polymerase (Transgen Biotech, Beijing, China). PCR products were confirmed by PCR analysis using 2×*EasyTaq^®^ PCR SuperMix* (Transgen Biotech, Beijing, China). Genomic DNA samples were prepared using the CTAB isolation buffer at pH 8.0 (20 g/L cetyltrimethylammonium bromide, 1.4 M sodium chloride, and 20 mM EDTA) [25]. The gene-specific primers are listed in Table S1. UV spectra were recorded on a Beckman DU 640 spectrophotometer (Beckman Coulter Inc., Brea, CA, USA). Specific optical rotations were obtained using a JASCO P-1020 digital polarimeter (JASCO Corporation, Tokyo, Japan). Electrospray ionization-mass spectrometry (ESIMS) and HRESIMS were obtained on a Thermo Scientific LTQ Orbitrap XL mass spectrometer (Thermo Fisher Scientific, Waltham, MA, USA) or using a Micromass Q-TOF ULTIMA GLOBAL GAA076 LC Mass spectrometer (Wasters Corporation, Milford, MA, USA). ^1^H NMR, ^13^C NMR and 2D NMR spectra were recorded on an Agilent 500 MHz DD2 spectrometer (Agilent Technologies Inc., Santa Clara, CA, USA). LC-MS was performed using an Acquity UPLC H-Class coupled to a SQ Detector 2 mass spectrometer using a BEH C18 column (1.7 μm, 2.1 × 50 mm, 1 mL/min) (Waters Corporation, Milford, MA, USA). Semi-preparative HPLC (YMC Co., Ltd., Kyoto, Japan) was performed on an ODS column (YMC-Pack ODS-A, 10 × 250 mm, 5 μm, 3 mL/min). Medium-pressure liquid chromatography (MPLC) was performed on a Bona-Agela CHEETAHTM HP100 (Beijing Agela Technologies Co., Ltd., Beijing, China) [26]. 

### 3.2. Materials and Culture Conditions

The fungal strain, authenticated as *P. dipodomyis* YJ-11, was collected from the marine sediment around the sewage outlet in Jiaozhou Bay of Qingdao. The strain was identified by internal transcribed spacer (ITS) sequence and the sequence data were submitted to GenBank (accession number: MK682303). Working stocks were prepared on Potato Dextrose agar slants stored at 4 °C in our laboratory.

The strain was incubated in media potato dextrose agar (PDA, 20% potato, 2% dextrose, and 2% agar) at 28 °C for 5 days for sporulation. Media potato sorbitol agar (PSA, 20% potato, 2% dextrose, 1.2 M D-sorbitol, and 2% agar) and PDA with 400 μg/mL zeocin (Sigma) were used to screen resistant transformants. The mutants were also cultured at 28 °C in an incubator. For compound production, the strains were cultured on potato dextrose (PDB, 20% potato and 2% dextrose) at 28 °C, 180 rpm for 9 days. *Trans*1-*T*1 Phage Resistant Chemically competent cell (Transgen Biotech, Beijing, China) was used for plasmid preservation and amplification, following standard recombinant DNA techniques. *E. coli* cultures were growing at 37 °C in another incubator. 

### 3.3. Sequence Analysis of the PdLaeA Gene

The LaeA gene was analyzed by Localblast with the reported LaeA obtained in national center for biotechnology information (NCBI). For the multiple sequence alignment analysis, the amino acid sequences of PdLaeA and other LaeA homologues from different species retrieved from NCBI were aligned using the ClustalX software [27]. The phylogenetic analysis was conducted with the MEGA7 software [28]. The conserved domain of the PdLaeA protein was scanned by the InterProScan program [29].

### 3.4. Construction of the PdLaeA Expression Vector

The overexpression vector pZeo which mainly contains a constitutive promoter PgpdA, resistant ampicillin gene and resistant zeocin gene as selection markers was digested with restriction endonucleases XhoI and XbaI. The PdLaeA gene was PCR-amplified (from genomic DNA of the wild-type strain *P. dipodomyis* YJ-11) using specific primers containing XhoI and XbaI restriction sites (Table S1). The PCR products were digested with the same endonucleases and PdLaeA gene was introduced into pZeo vector to generate pZeo-PdLaeA (Figure S3 in Supplementary Materials). The recombinant vector was transformed into competent *E. coli* strain *Trans*1-*T*1 to extract plasmids for transformation.

### 3.5. Fungal Protoplast Formation and Transformation

The strain *P. dipodomyis* YJ-11 was first grown on PDA plates at 28 °C for 5 days. Fresh spores were collected into 50 mL PDB together with yeast extract media in 250 mL Erlenmeyer flasks and germinated at 28 °C and 180 rpm for about 7 h. Mycelia were gathered by centrifugation at 4000 rpm for 15 min, and washed by 25 mL osmotic buffer (1.2 M MgSO_4_, 10 mM sodium phosphate, pH 5.8). Subsequently, the mycelia were suspended into 10 mL of osmotic buffer containing 30 mg lysing enzymes from *Trichodema harzianum* (Sigma) and 20 mg Yatalase (TaKaRa), transferred into an empty sterile bottle, and cultured in a shaker of 28 °C at 80 rpm overnight to form protoplast. 

After the whole night, the mixture was collected in a 50 mL centrifuge tube and covered gently with isopyknic protoplast trapping buffer (0.6 M sorbitol, 0.1 M pH 7.0 Tris-HCl). After centrifugation at 4000 rpm for 15 min at 4 °C, protoplasts were collected in the interface of the above two buffers. The protoplasts were then transferred to a sterile 50 mL centrifuge tube and washed by 20 mL STC buffer (1.2 M sorbitol, 10 mM CaCl2, 10 mM pH 7.5 Tris-HCl). The protoplasts were resuspended in 2 mL STC buffer for transformation. Then, the freeze-drying plasmids pZeo-PdLaeA and the plasmid pZeo (the desired mutants were regarded as a control in the following crude analysis) were dissolved in 50 μL STC buffer, followed by 100 μL protoplast suspension and the mixture incubated for 60 min on ice. Next, 600 μL of polyethylene glycol (PEG) solution (60% PEG, 50 mM calcium chloride and 50 mM pH 7.5 Tris-HCl) was added to the protoplast mixture, and the mixture was incubated at room temperature for an additional 25 min. The mixture was spread on the regeneration solid PSA medium (PDA medium with 1.2 M sorbitol and 400 μg/mL zeocin) and incubated at 28 °C for around 4 days [30]. 

### 3.6. Transformants Screening

After regeneration, the transformants were passaged to PDA plates with 400 μg/mL zeocin respectively. Zeocin resistant mutants were transferred onto new PDA media containing 400 μg/mL zeocin for the second screening. The strains that were able to grow were subjected to further PCR analysis validation. The putative OE::PdLaeA mutants and the wild-type strain were cultured on PDA media for 5 days at 28 °C in an incubator in order to extract genomic DNAs. PCR analysis to confirm the gene insertion was carried out using three pairs of primers to verify the zeocin resistant mutants as shown in Table S1 and Figure S4 (primers gpda-1 and gpda-2 to verify whole of the PdLaeA gene, primers gpda-1 and YZ-LaeA-F to verify the upstream of target gene, primers gpda-2 and YZ-LaeA-R to verify the downstream of target gene). Those transformants which went through all these verifications were recognized as the desired mutants.

### 3.7. Fermentation and Extraction

For small-scale analysis, the OE::PdLaeA mutant strains and the control strain were grown on PDA plates for 5 days at 28 °C. Shortly after sporing, they were inoculated into 150 mL of PDB medium and cultured at 28 °C, 180 rpm. Nine days later, the cultures were extracted with twice the volume of ethyl acetate. The organic phase was evaporated and the residue was dissolved in MeOH, which was analyzed by HPLC and indicated that one of the six mutants showed an apparent change in metabolite production (Figure 1). 

For compound isolation, the selected strain was initially handled the same as above. Then a large-scale fermentation was performed in 500 mL Erlenmeyer flasks (total 10 L) for further incubation. The broth was extracted three times with ethyl acetate to give a total of 60 L of extract solution. The organic phase was evaporated under reduced pressure to afford a crude residue (12 g).

### 3.8. Compound Isolation

The crude extracts were separated by MPLC (60% to 100% MeOH in H_2_O for 60 min). The fractions containing the target compounds were combined and concentrated to give 7 fractions (Fr.1 to Fr.7). For further purification, semi-preparative HPLC were carried out. Fr.4 was then purified by HPLC (55% MeCN in H_2_O, 0.5% THF) to obtain Fr.4-1 (**6**; 5 mg; t_R_ 24.2 min). Fr.5 was purified by HPLC (42% MeCN in H_2_O, 0.5% THF) to obtain Fr.5-1 (**3**;18 mg; t_R_ 30.8 min) and Fr.5-2 (**1**; 7 mg; t_R_ 38.0 min). Fr.6 was purified by HPLC (60% MeOH in H_2_O, 0.5% THF) to give five subfractions (Fr.6- 1 to Fr.6-5), in which Fr.6-1 (**1**; 4 mg; t_R_ 30.3 min), Fr.6-4 (**2**; 35 mg; t_R_ 46.1 min), Fr.6-5 (**5**; 6 mg; t_R_ 52.2 min) were proven to be pure. Fr.6-3-1 (**4**; 84 mg; t_R_ 20.5 min) was purified by HPLC (50% MeCN in H_2_O, 0.5% THF) from Fr.6-3. The purity of each compound was checked by LC-MS and the structures were confirmed by NMR including ^1^H, ^13^C, and 2D NMR spectra. 

10,11-Dihydrobislongiquinolide (**1**): ${\left[\alpha \right]}_{D}^{20}$ 35.6 (c 0.13, MeOH), UV (MeOH) *λ*_max_ (log ε): 228 (3.67), 296 (2.97) nm, ^1^H-NMR (CDCl_3_, 500 MHz) and ^13^C-NMR (CDCl_3_, 125 MHz) data are shown in Table 1 and Table 2, HRESIMS *m*/*z*: 497.2185 [M − H]^−^ (Calcd. for C_28_H_34_O_8_: 497.2170). 

10,11,16,17-Tetrahydrobislongiquinolide (**2**): ${\left[\alpha \right]}_{D}^{20}$ 71.8 (c 0.50, MeOH), UV (MeOH) *λ*_max_ (log ε): 237 (1.22), 296 (3.24) nm, ^1^H-NMR (CDCl_3_, 500 MHz) and ^13^C-NMR (CDCl_3_, 125 MHz) data are shown in Table 1 and Table 2, HRESIMS *m*/*z*: 499.2343 [M − H]^−^ (Calcd. for C_28_H_36_O_8_: 499.2326).

### 3.9. Hydrogenation of Compounds 1–4

Compounds **1**–**4** in methanol were hydrogenated over palladium on carbon at room temperature overnight, respectively. The reaction mixture was filtered, concentrated, and purified by semi- preparative HPLC (65% MeOH in H_2_O, 0.1% THF) to give octahydrobislongiquinolide (**7**; 5 mg; t_R_ 15.6 min). The purity of compound **7** was checked by LC-MS and the structures were confirmed by NMR including ^1^H, ^13^C and 2D NMR spectra.

Octahydrobislongiquinolide (**7**): ${\left[\alpha \right]}_{D}^{20}$ 78.4 (c 0.10, MeOH), UV (MeOH) *λ*_max_ (log ε): 227 (4.78), 297 (2.13) nm, ^1^H-NMR (CDCl_3_, 600 MHz) and ^13^C-NMR (CDCl_3_, 150 MHz) data are shown in Table 1 and Table 2, HRESIMS *m*/*z*: 527.2608 [M + Na]^+^ (calcd. 527.2615 for C_28_H_40_O_8_Na).

### 3.10. Assay of Cytotoxicity, Antimicrobial and Antioxidation Activity

These biological evaluations were carried out as previously reported in References [26,31]. 

### 3.11. Assay of Siderophore Activity

Siderophore activity was evaluated by the chrome azurol sulfonate (CAS) assay. Buffer A consist of 50 mL 5 M CAS solution and 10 mL Fe^3+^ solution (1 mM FeCl_3_·6H_2_O, 10 mM HCl). 72.9 mg hexadecyl trimethyl ammonium bromide (HDTMA) was dissolved in 40 mL water after heated as buffer B. Buffer B was then slowly added to buffer A under stirring to afford 100 mL CAS assay solution. All tested compounds were dissolved in MeOH at stepwise concentrations (1–100 mM) then 99 µL of the compound solution and 1 µL CAS assay solution were dispensed into wells of a 96-well microtiter tray. The mixture was shaken and left to stand for 4 h. After that, absorbance was measured at 630 nm and the inhibition rate was calculated. Deferoxamine mesylate, which could make the color change of CAS from blue to orange, was used to determine standard curves relating the CAS reactivity to iron-binding ligands. The ED_50_ values denoted the concentration of sample required to remove 50% of the iron in CAS solution [32,33]. 

## 4. Conclusions

In summary, guided by bioinformatic analysis of the genomic sequence of *P. dipodomyis* YJ-11, we discovered one global regulator of PdLaeA. Further overexpression of the PdLaeA gene in *P. dipodomyis* YJ-11 led to the discovery of two new compounds, 10,11-dihydrobislongiquinolide (**1**), 10,11,16,17-tetrahydrobislongiquinolide (**2**), together with four known sorbicillin analogues (compounds **3**–**6**). This is the first report on application of global regulator LaeA in *P. dipodomyis* with the purpose of increasing the secondary metabolite producing potential. This result also indicated that the production of sorbicillinoids may be regulated by the global regulator LaeA. 

## Figures and Tables

**Table 1:** ^1^H NMR data of experimental compounds **1** and **2** (500 MHz, CDCl_3_, TMS, δ ppm), literature shared compound **3** [16] (400 MHz, CDCl_3_, δ ppm), and the hydrogenation product **7** (600 MHz, CDCl_3_, TMS, δ ppm) (*J* in Hz).

No.	1	2	3 [16]	7
1	--	--	--	--
2	--	--	--	--
3	--	--	--	--
4	3.36 s	3.42 s	3.36 br. s	3.39 s
5	--	--	--	--
6	--	--	--	--
7	2.95 d (7.0)	2.65 d (6.6)	3.43 d (4.8)	2.75 d (7.3)
8	3.20 br. d (7.0)	3.15 br. d (6.6)	3.21 dd (1.2, 4.8)	3.08 br. d (7.3)
9	--	--	--	--
10	2.51 t (7.4)	2.45-2.60^a^	6.12 d (15.0)	2.40–2.50 ^a^
11	2.42 m	2.39 m	7.33 dd (15.0, 10.6)	1.70 m
12	5.44 dt (15.3, 6.7)	5.44–5.55 ^a^	6.28 dd (14.4, 10.6)	1.30–1.40 ^a^
13	5.52 dq (15.3, 6.0)	5.44–5.55 ^a^	6.24 dq (14.4, 6.8)	1.30–1.40 ^a^
14	1.64 d (6.0)	1.59–1.67 ^a^	1.88 d (6.8)	0.87 t (7.3)
15	--	--	--	--
16	6.02 d (15.0)	2.45–2.60 ^a^	6.13 d (15.0)	2.40–2.50 ^a^
17	7.16 dd (15.0, 10.6)	2.11, 2.19 m	7.22 dd (15.0, 10.4)	1.48 m
18	6.24 dd (15.0, 10.6)	5.34 m	6.21 dd (15.2, 10.4)	1.20–1.30 ^a^
19	6.42 m	5.44–5.55 ^a^	6.38 dq (15.2, 7.2)	1.20–1.30 ^a^
20	1.93 d (6.7)	1.59–1.67 ^a^	1.90 d (7.2)	0.89 t (6.5)
21	--	--	--	--
22	--	--	--	--
23	--	--	--	--
24	--	--	--	--
1-CH_3_	1.05 s	1.13 s	1.12 s	1.11 s
5-CH_3_	1.34 s	1.31 s	1.29 s	1.30 s
21-CH_3_	1.57 s	1.55 s	1.51 s	1.50 s
23-CH_3_	1.61 s	1.71 s	1.58 s	1.67 s
9-OH	14.45 br. s	14.45 br. s	13.99 br. s	14.46 br. s

^a^ Overlapped by other signals.

**Table 2:** ^13^C NMR data of experimental compounds **1** and **2** (125 MHz, CDCl_3_, TMS, δ ppm), literature shared compound **3** [16] (100 MHz, CDCl_3_, δ ppm) and the hydrogenation product **7** (150 MHz, CDCl_3_, TMS, δ ppm).

No.	1	2	3 [16]	7
1	61.7	61.4	62.6	61.4
2	193.0	192.9	194.9	192.9
3	108.4	108.4	108.5	106.3
4	42.8	42.7	42.4	42.9
5	75.3	75.2	75.0	75.3
6	209.9	209.1	208.3	209.2 ^b^
7	50.7	54.0	51.3	54.3
8	42.0	42.6	43.6	42.7
9	181.3	181.2	169.8	182.4
10	32.5	32.4	117.6	32.4
11	28.4	28.4	143.9	25.2
12	129.1	129.0	130.3	22.5
13	126.9	126.9	140.9	31.9
14	17.8	17.8	18.9	14.0
15	202.1	213.2	202.7	214.3
16	127.2	45.9	127.0	46.0
17	148.3	25.9	148.0	22.5
18	130.1	128.5	131.0	31.0
19	146.0	126.8	145.5	29.8
20	19.2	17.8	19.1	14.0
21	83.1	84.0	83.2	83.1
22	175.8	177.3	176.5	176.0
23	98.8	97.8	98.2	98.2
24	174.4	175.4	174.9	173.6
1-CH_3_	10.5	10.6	11.0	10.7
5-CH_3_	24.1	24.0	23.5	24.2
21-CH_3_	21.8	21.3	23.1	21.5
23-CH_3_	6.1	5.9	6.3	6.1

^b^ Detected by HMBC spectrum.

## Supplementary Materials

The following are available online at https://www.mdpi.com/1660-3397/17/8/446/s1, Figure S1: AntiSMASH analysis of the genome of the strain *Penicillium dipodomyis* YJ-11, Figure S2: Phylogenetic tree analysis of PdLaeA and its homologs from different species, Figure S3: Map of the vector pZeo and PdLaeA overexpression plasmid pZeo-PdLaeA, Figure S4: PCR analysis for confirming the gene insertion, Figures S5–S11: 1D, 2D NMR and HRESIMS spectra of 10,11-dihydrobislongiquinolide (**1**), Figures S12–S18: 1D, 2D NMR and HRESIMS spectra of 10,11,16,17-tetrahydrobislongiquinolide (**2**). Figures S19–S20: 1D NMR spectra of bislongiquinolide (**3**). Figures S21–S22: 1D NMR spectra of 16,17-dihydrobislongiquinolide (**4**), Figures S23–S28: 1D, 2D NMR and HRESIMS spectra of octahydrobislongiquinolide (**7**), Table S1: The primers used in this study.
