# Supplementary material for: Discovery of Two New Sorbicillinoids by Overexpression of the Global Regulator LaeA in a Marine-Derived Fungus Penicillium dipodomyis YJ-11
Source: Mar Drugs. 2019 Jul 28;17(8):446. doi: 10.3390/md17080446 (PMC6723206; doi:10.3390/md17080446)

Supporting Information

Discovery of Two New Sorbicillinoids by Overexpression of the Global Regulator LaeA in a Marine-derived Fungus *Penicillium dipodomyis* YJ-11

Jing Yu [1], Huan Han [1], Xianyan Zhang [1], Chuanteng Ma [1], Chunxiao Sun [1], Qian Che [1], Qianqun Gu [1], Tianjiao Zhu [1,2], Guojian Zhang [1,2] and Dehai Li [1,2,*]

Table S1. The primers used in this study. (5' to 3') 2
Figure S1. AntiSMASH analysis of the genome of the strain *Penicillium dipodomyis* YJ-11. The predicted 45 gene clusters include 15 PKS, 10 NRPS, 4 Terpene, 1 Indole, 2 PKS-NRPS hybrids, 1 Indole-NRPS hybrid, 1 Indole-Terpene hybrid and 11 Other clusters. 2
Figure S2. Phylogenetic tree analysis of PdLaeA and its homologs from different species. Branch lengths are in proportion to distance. 3
Figure S3. Map of the vector pZeo and PdLaeA overexpression plasmid pZeo-PdLaeA. 3
Figure S4. PCR analysis for confirming the gene insertion. The results showed that mutants 1-5 were desired. 4
Figure S5. ^{1}H NMR (500 MHz, $CDCl_3$) spectrum of compound **1**. 4
Figure S6. ^{13}C NMR (125 MHz, $CDCl_3$) spectrum of compound **1**. 5
Figure S7. HSQC (500 MHz, $CDCl_3$) spectrum of compound **1**. 6
Figure S8. ^{1}H-^{1}H COSY (500 MHz, $CDCl_3$) spectrum of compound **1**. 6
Figure S9. HMBC (500 MHz, $CDCl_3$) spectrum of compound **1**. 7
Figure S10. NOESY (500 MHz, $CDCl_3$) spectrum of compound **1**. 7
Figure S11. HRESIMS spectrum of compound **1**. 8
Figure S12. ^{1}H NMR (500 MHz, $CDCl_3$) spectrum of compound **2**. 8
Figure S13. ^{13}C NMR (125 MHz, $CDCl_3$) spectrum of compound **2**. 9
Figure S14. HSQC (500 MHz, $CDCl_3$) spectrum of compound **2**. 10
Figure S15. ^{1}H-^{1}H COSY (500 MHz, $CDCl_3$) spectrum of compound **2**. 10
Figure S16. HMBC (500 MHz, $CDCl_3$) spectrum of compound **2**. 11
Figure S17. NOESY (500 MHz, $CDCl_3$) spectrum of compound **2**. 11
Figure S19. ^{1}H NMR (500 MHz, $CDCl_3$) spectrum of compound **3**. 12
Figure S20. ^{13}C NMR (125 MHz, $CDCl_3$) spectrum of compound **3**. 13
Figure S21. ^{1}H NMR (500 MHz, $CDCl_3$) spectrum of compound **4**. 13
Figure S22. ^{13}C NMR (125 MHz, $CDCl_3$) spectrum of compound **4**. 14
Figure S23. ^{1}H NMR (600 MHz, $CDCl_3$) spectrum of compound **7**. 14
Figure S24. ^{13}C NMR (150 MHz, $CDCl_3$) spectrum of compound **7**. 15
Figure S25. HSQC (600 MHz, $CDCl_3$) spectrum of compound **7**. 15
Figure S26. ^{1}H-^{1}H COSY (600 MHz, $CDCl_3$) spectrum of compound **7**. 16
Figure S27. HMBC (600 MHz, $CDCl_3$) spectrum of compound **7**. 17
Figure S28. HRESIMS spectrum of compound **7**. 18

Table S1. The primers used in this study. (5' to 3')

Primers	Sequences
LaeA-F	CCGctcgagTTATTCCTCGACGGGTTTCCGG
LaeA-R	GCtctagaATGTTTACGAACGGGGATTCCAG
gpda-1	TACAGACAAGCTGTGACCGTCTC
gpda-2	CGTTAAGTGGATCTCGGTGACGG
YZ-LaeA-F	CACAATTGGGTACCAGGTGACATAC
YZ-LaeA-R	GATACAAGTCCGAGTCAACCCCAG

Note: The sites of the restriction endonucleases are represented by lowercase letters.

Figure S1. AntiSMASH analysis of the genome of the strain *Penicillium dipodomyis* YJ-11. The predicted 45 gene clusters include 15 PKS, 10 NRPS, 4 Terpene, 1 Indole, 2 PKS-NRPS hybrids, 1 Indole-NRPS hybrid, 1 Indole-Terpene hybrid and 11 Other clusters.

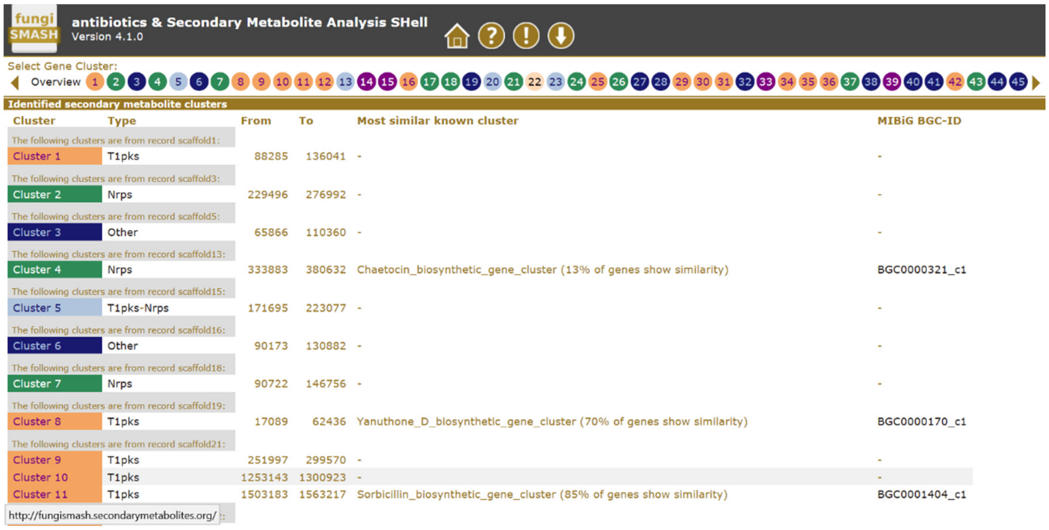

Figure S2. Phylogenetic tree analysis of PdLaeA and its homologs from different species. Branch lengths are in proportion to distance.

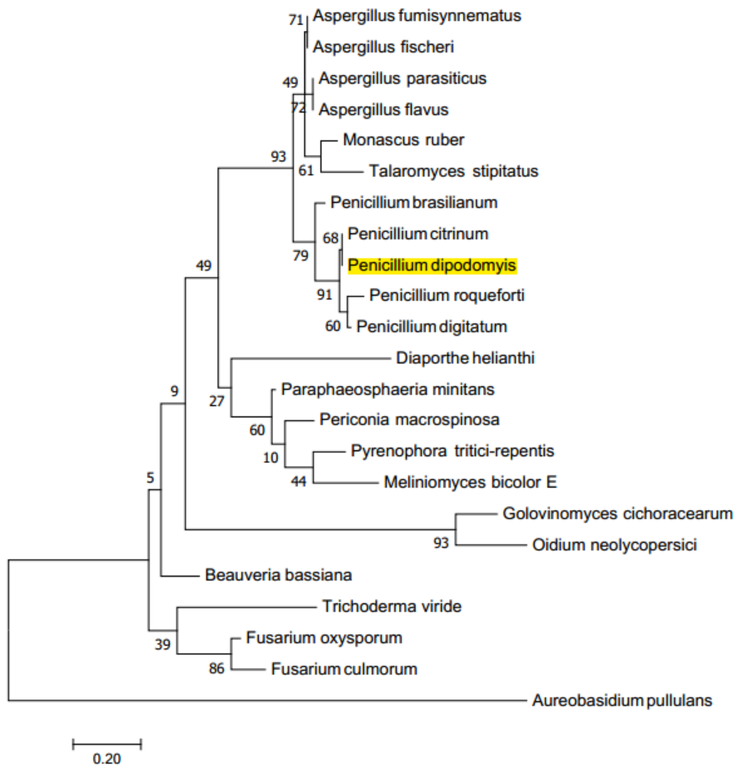

Figure S3. Map of the vector pZeo and PdLaeA overexpression plasmid pZeo-PdLaeA.

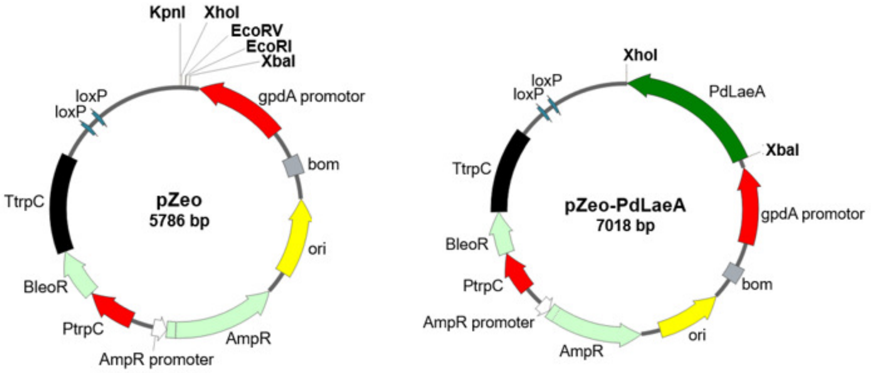

Figure S4. PCR analysis for confirming the gene insertion. The results showed that mutants 1-5 were desired.

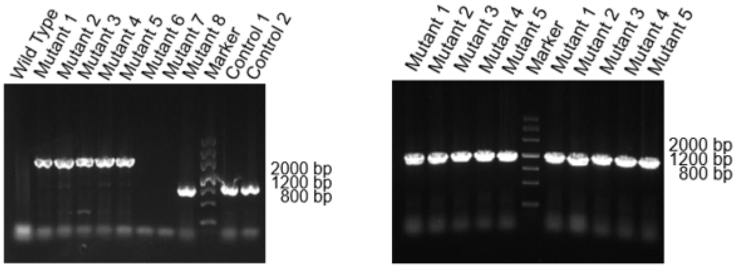

Figure S5. ^{1}H NMR (500 MHz, $CDCl_3$) spectrum of compound **1**.

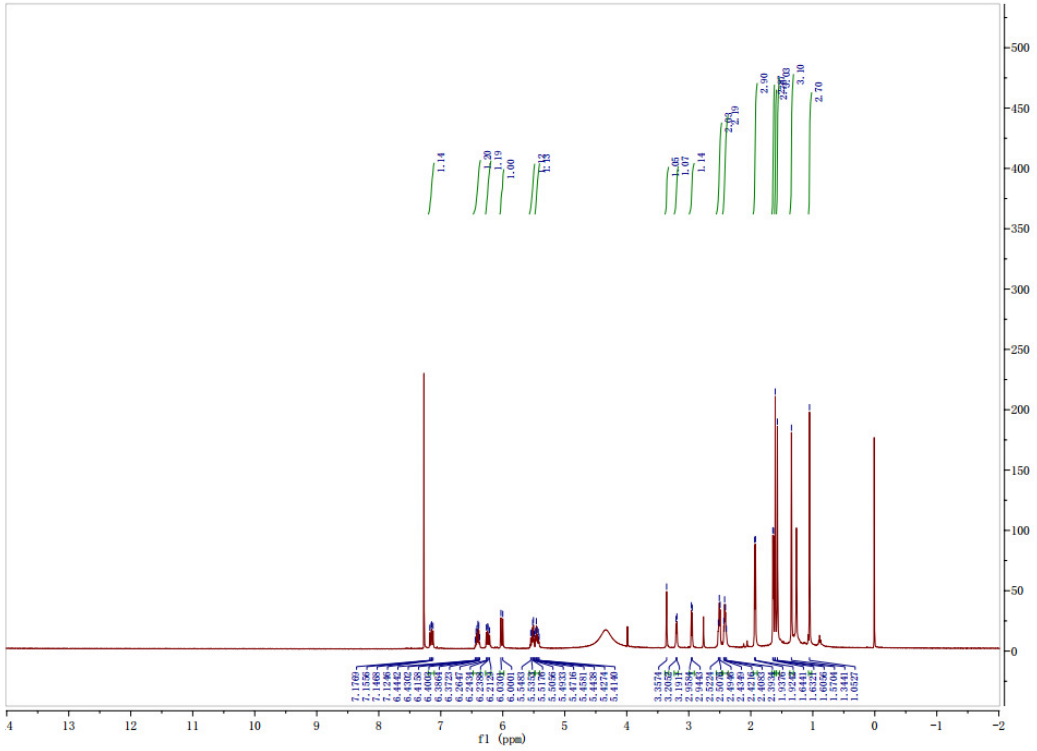

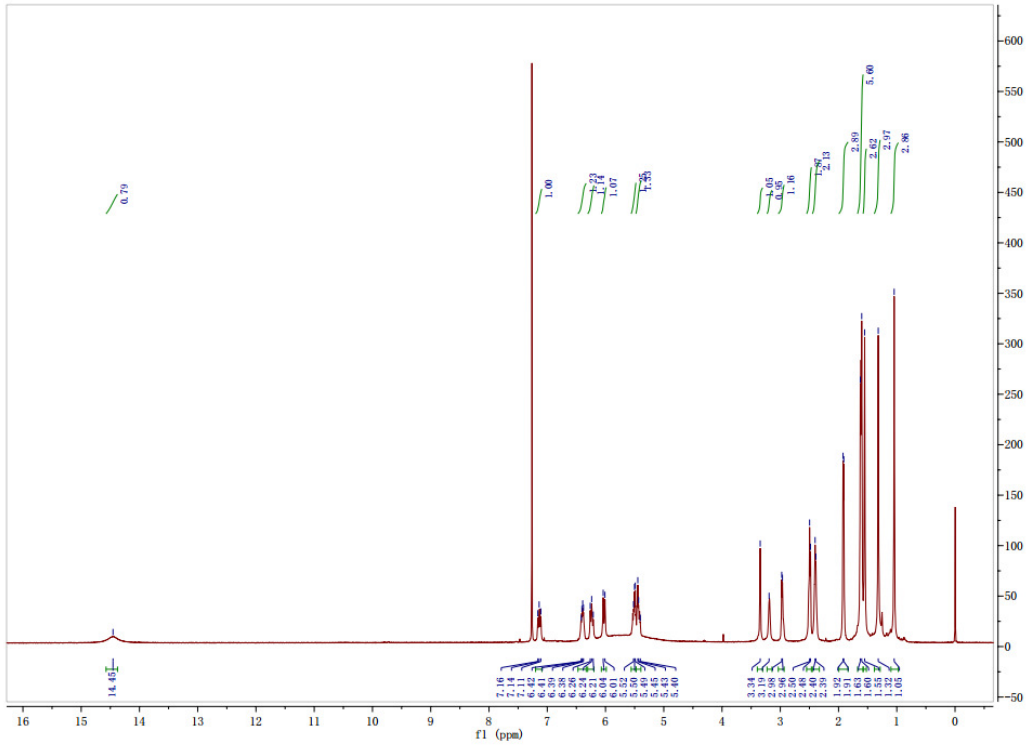

Figure S6. ^{13}C NMR (125 MHz, $CDCl_3$) spectrum of compound **1**.

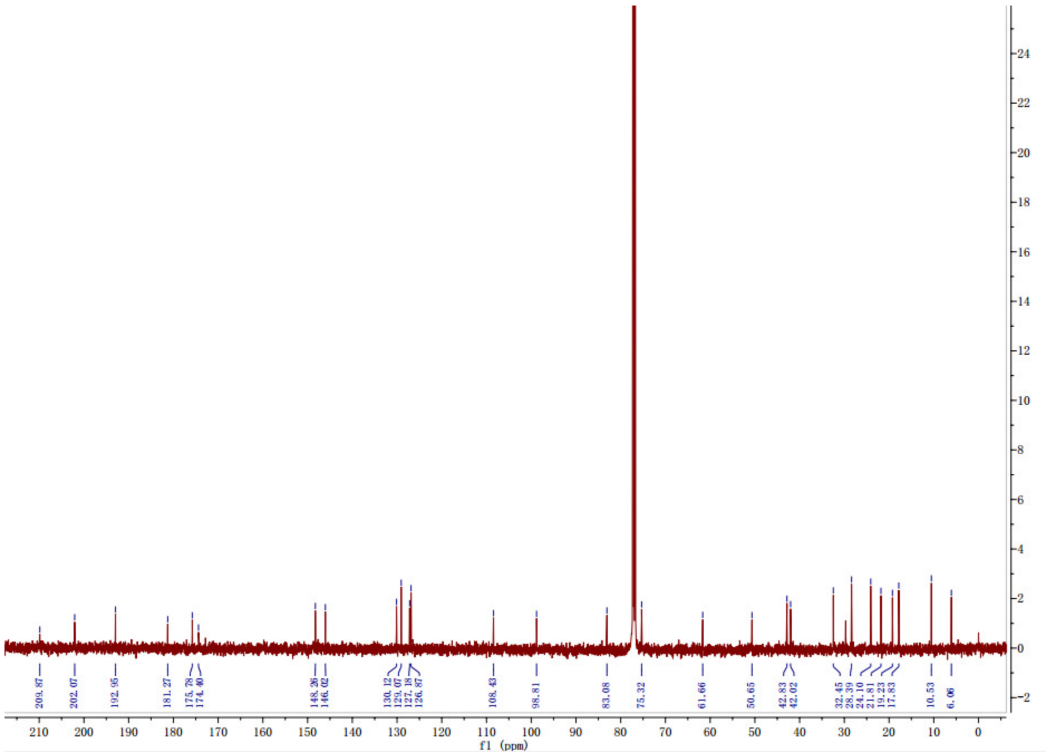

Figure S7. HSQC (500 MHz, $CDCl_3$) spectrum of compound **1**.

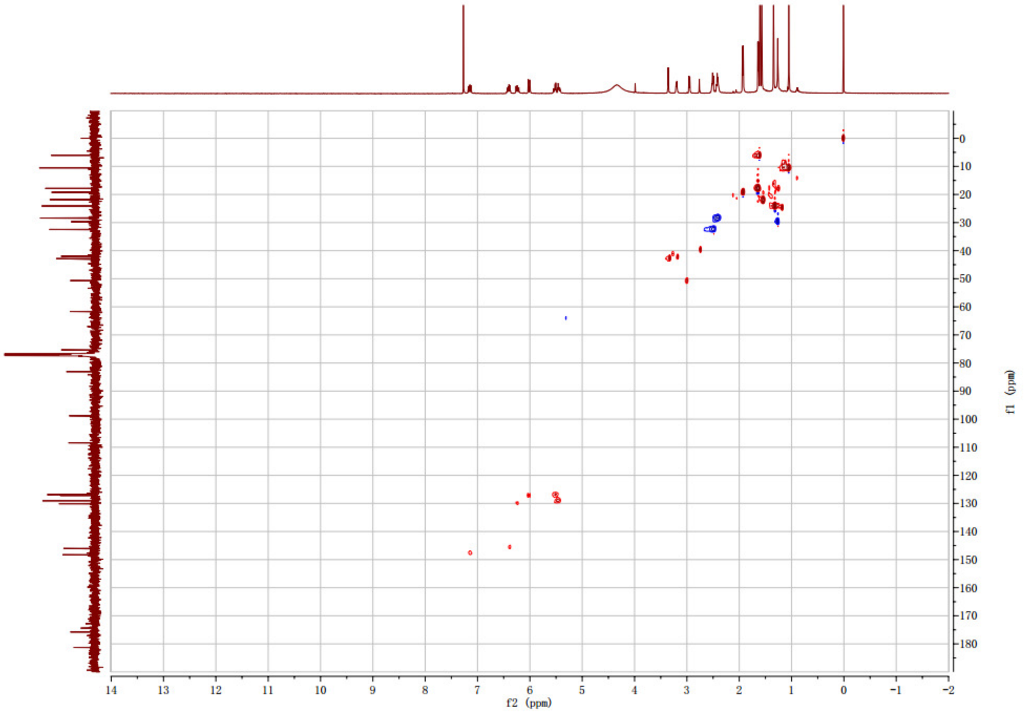

Figure S8. ^1H-^1H COSY (500 MHz, $CDCl_3$) spectrum of compound **1**.

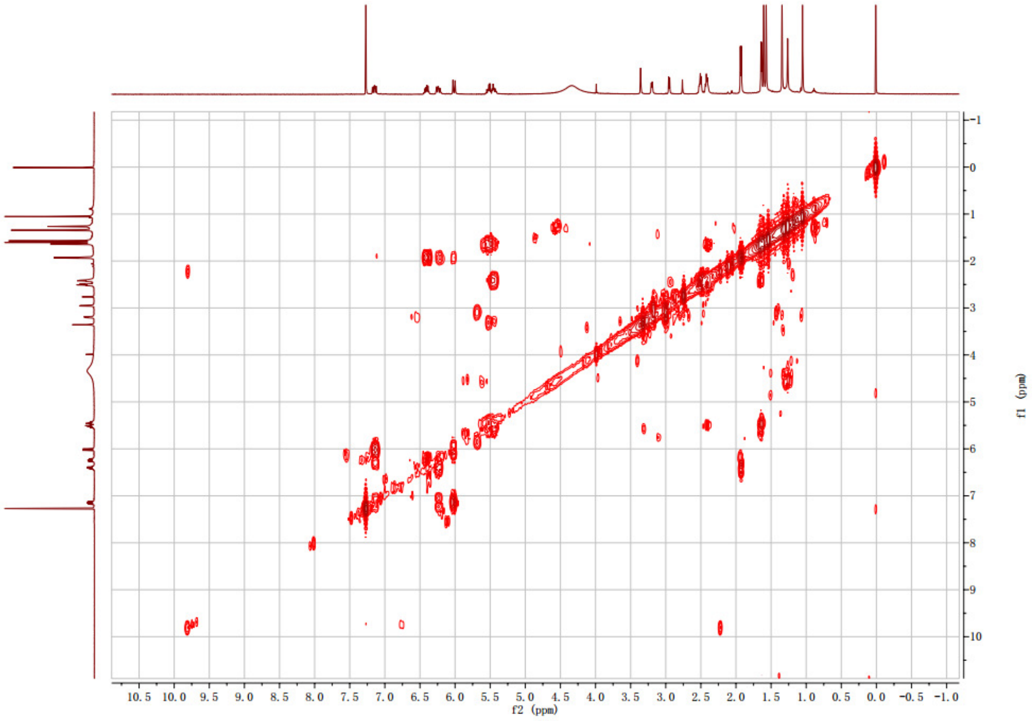

Figure S9. HMBC (500 MHz, CDCl3) spectrum of compound **1**.

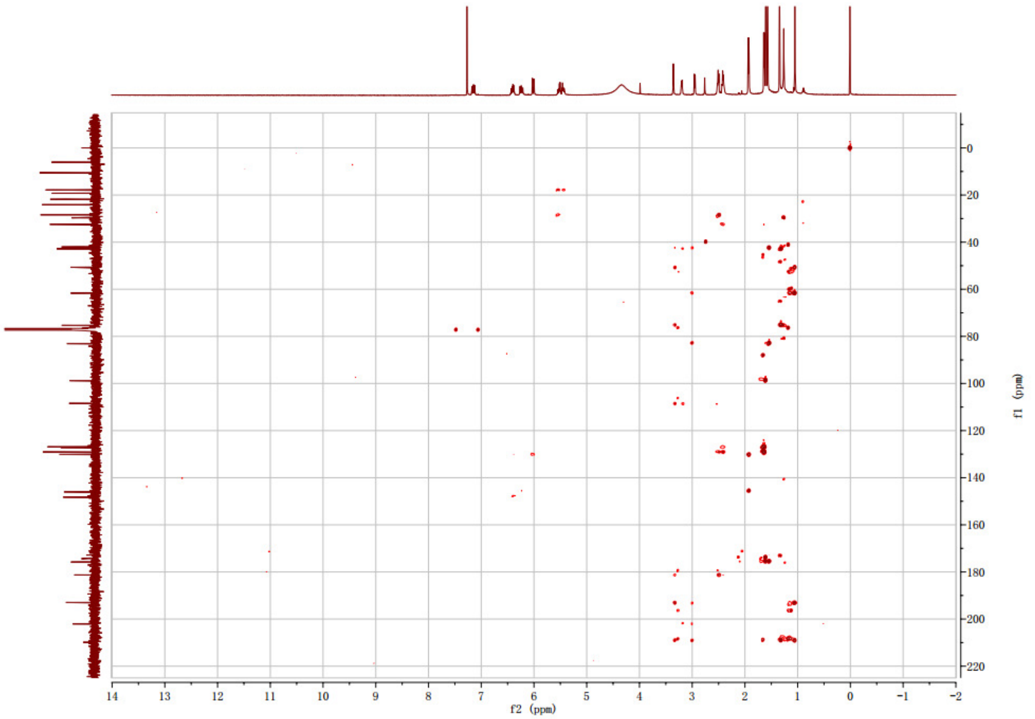

Figure S10. NOESY (500 MHz, CDCl3) spectrum of compound **1**.

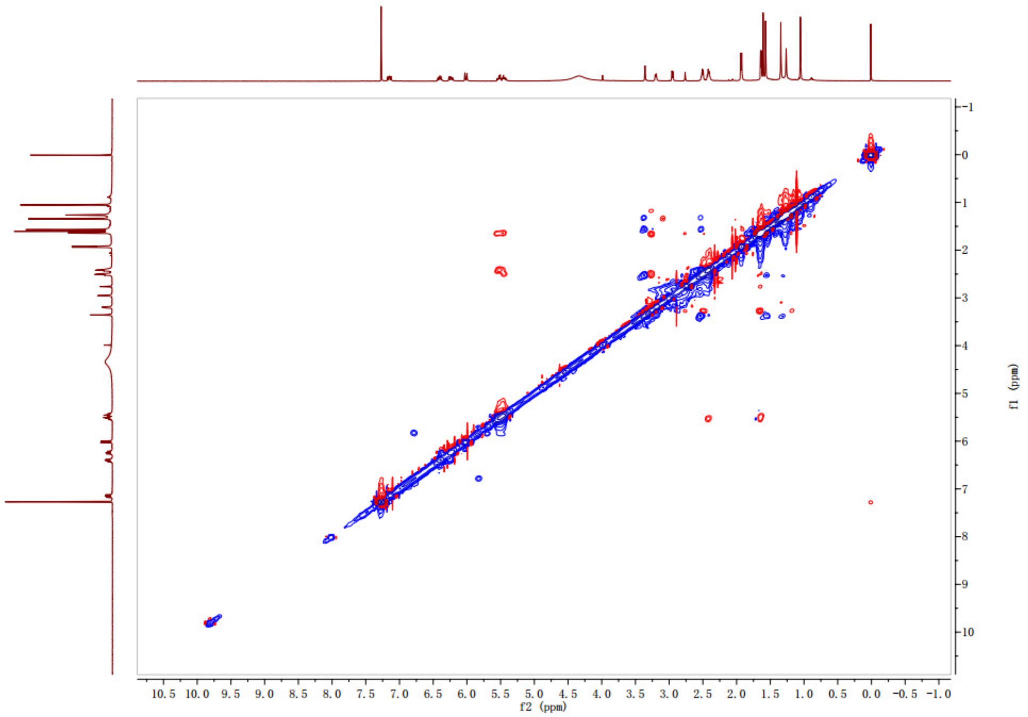

Figure S11. HRESIMS spectrum of compound **1**.

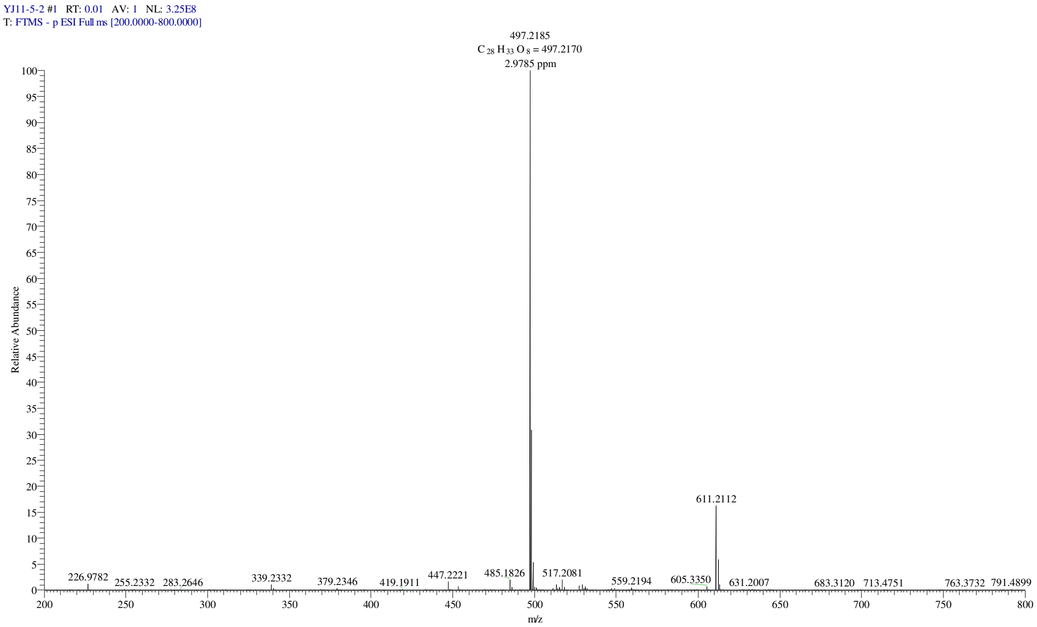

Figure S12. ^{1}H NMR (500 MHz, $CDCl_3$) spectrum of compound **2**.

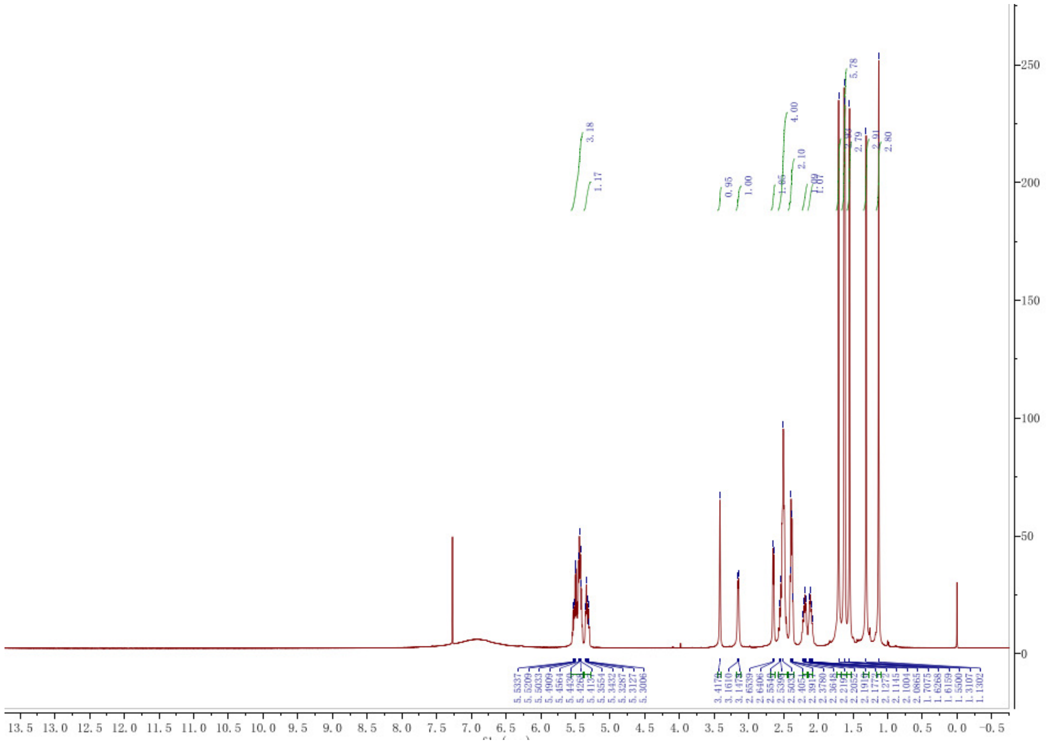

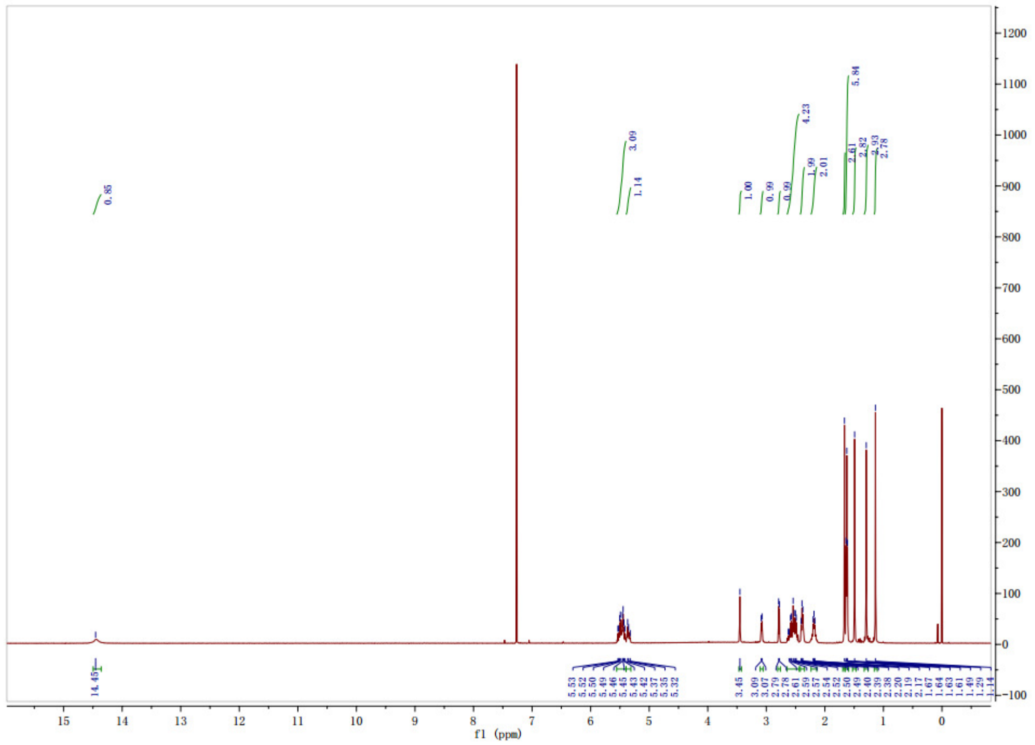

Figure S13. ^{13}C NMR (125 MHz, $CDCl_3$) spectrum of compound **2**.

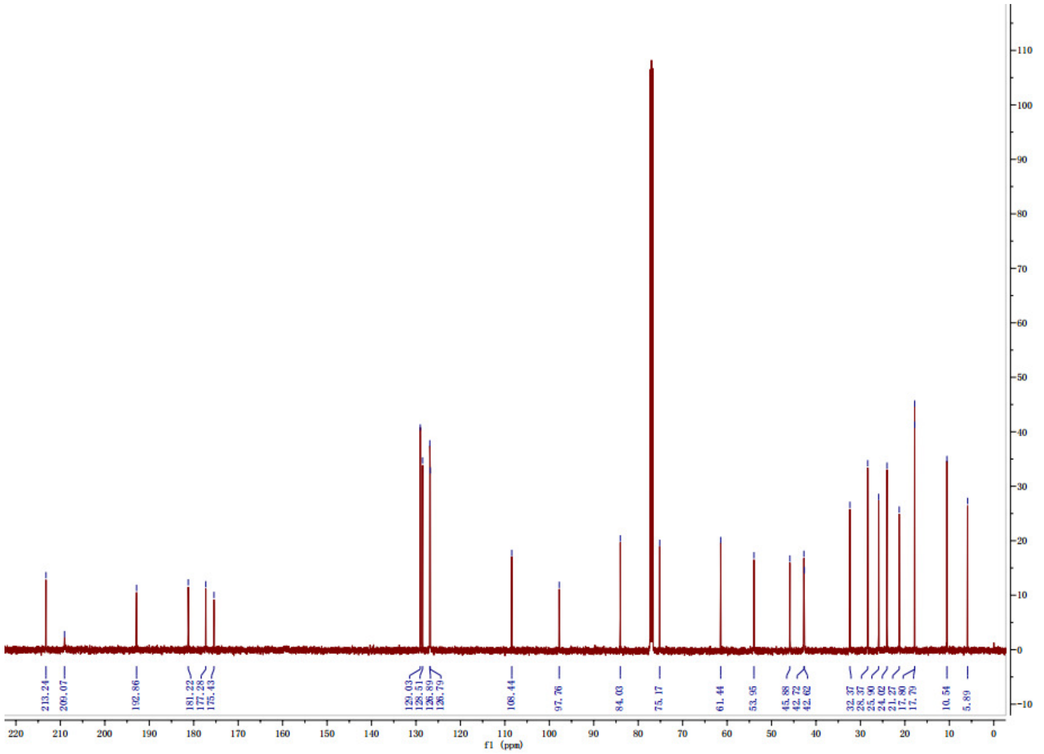

Figure S14. HSQC (500 MHz, $CDCl_3$) spectrum of compound **2**.

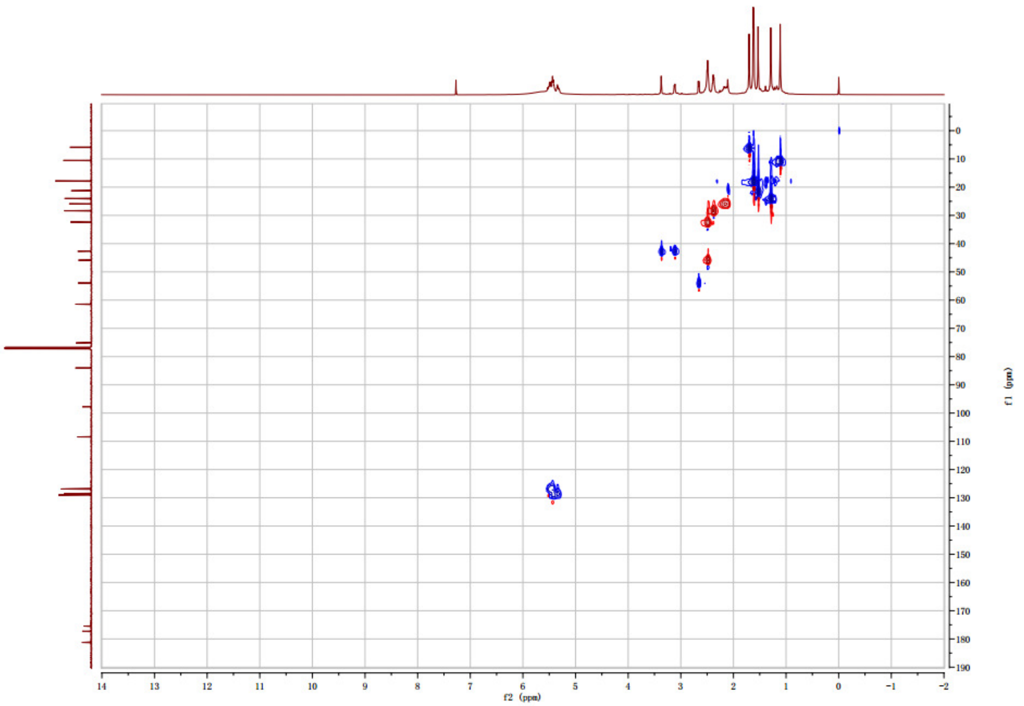

Figure S15. ^1H-^1H COSY (500 MHz, $CDCl_3$) spectrum of compound **2**.

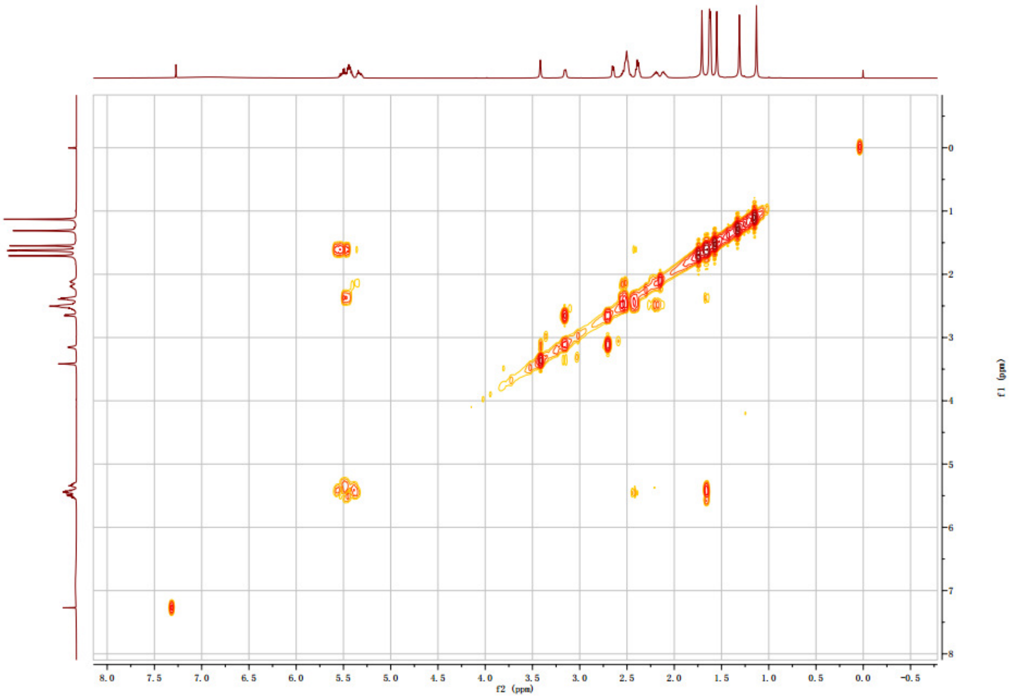

Figure S16. HMBC (500 MHz, $CDCl_3$) spectrum of compound **2**.

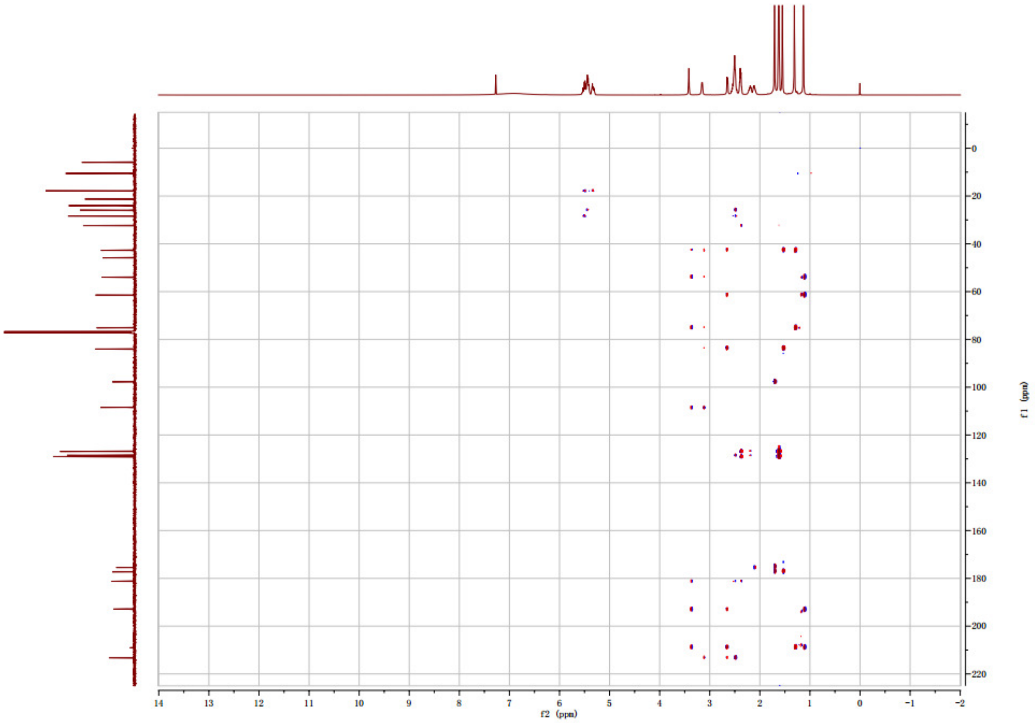

Figure S17. NOESY (500 MHz, $CDCl_3$) spectrum of compound **2**.

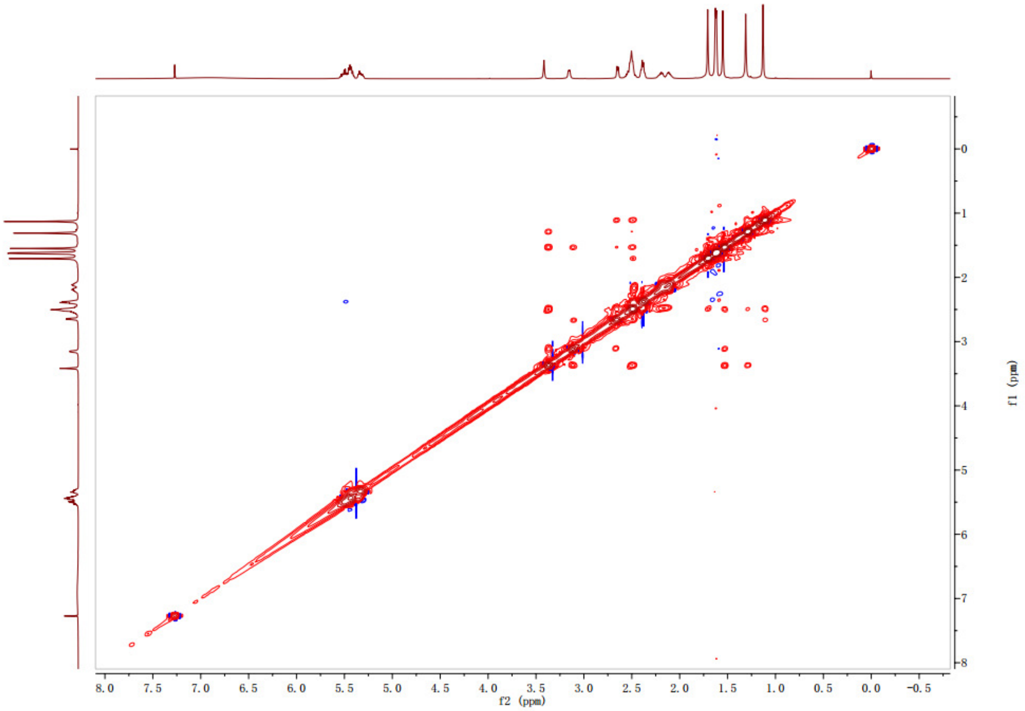

Figure S18. HRESIMS spectrum of compound **2**.

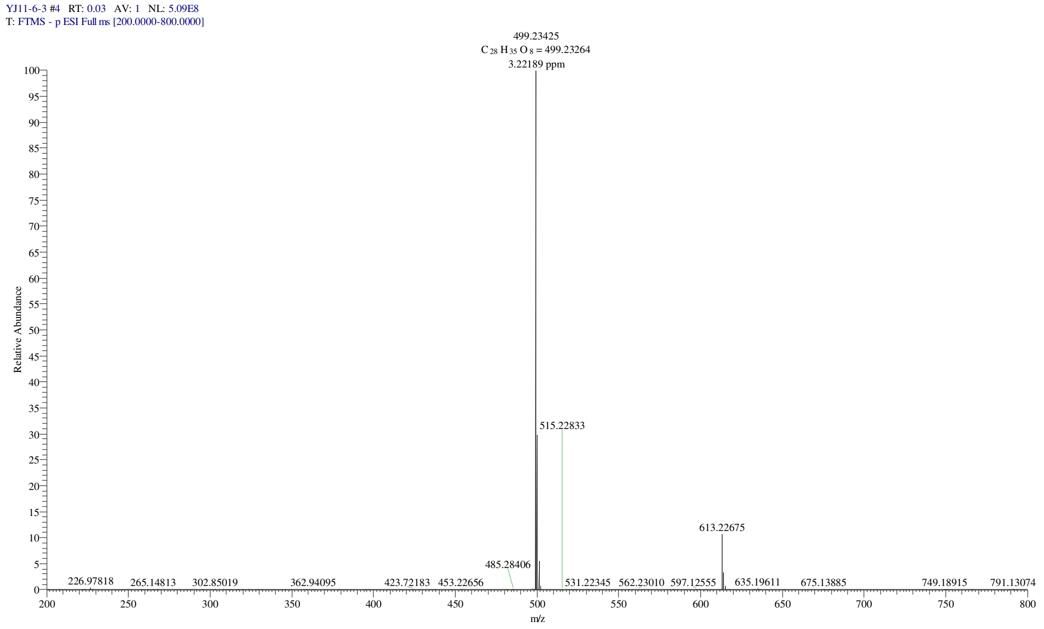

Figure S19. ^{1}H NMR (500 MHz, $CDCl_3$) spectrum of compound **3**.

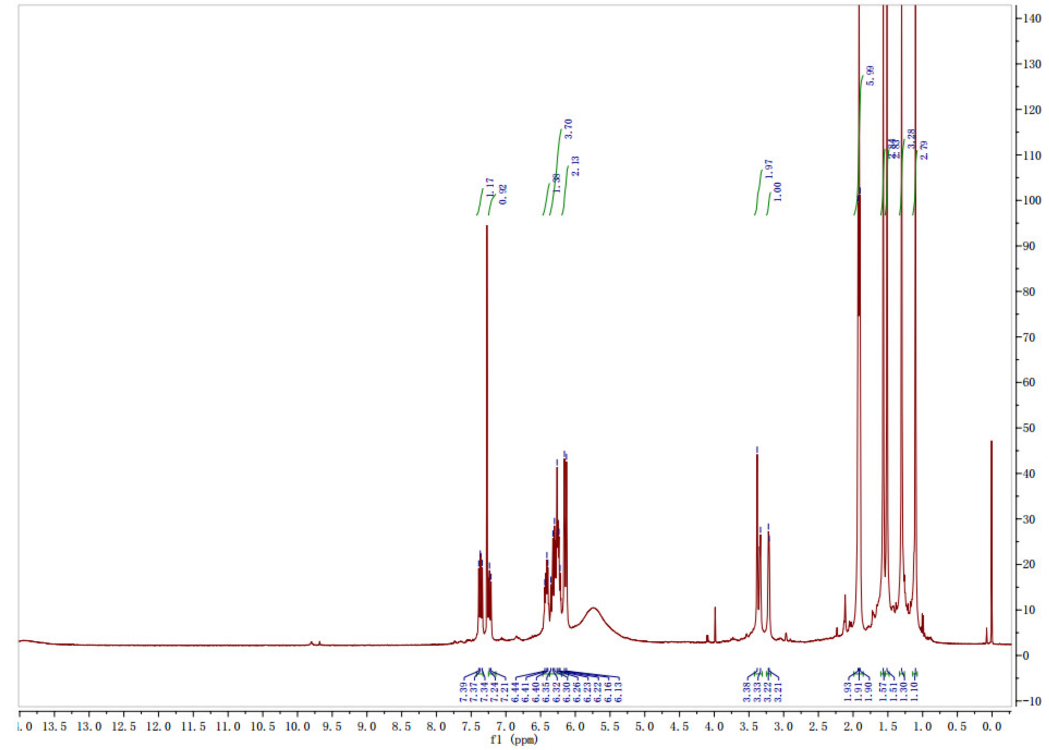

Figure S20. ^{13}C NMR (125 MHz, $CDCl_3$) spectrum of compound **3**.

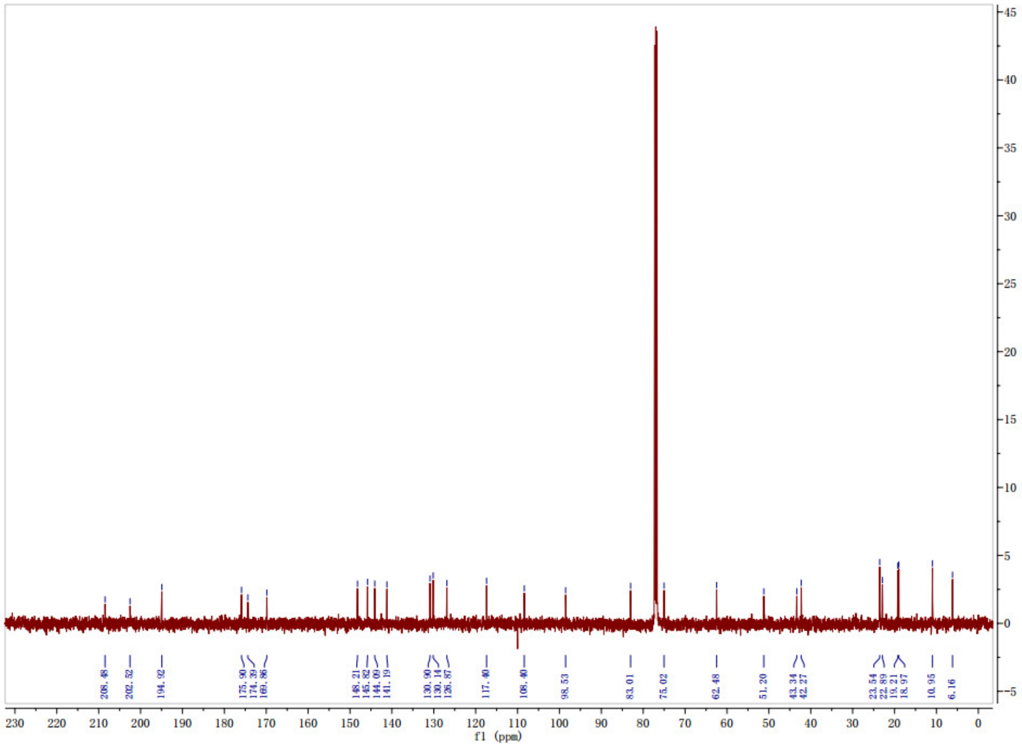

Figure S21. ^{1}H NMR (500 MHz, $CDCl_3$) spectrum of compound **4**.

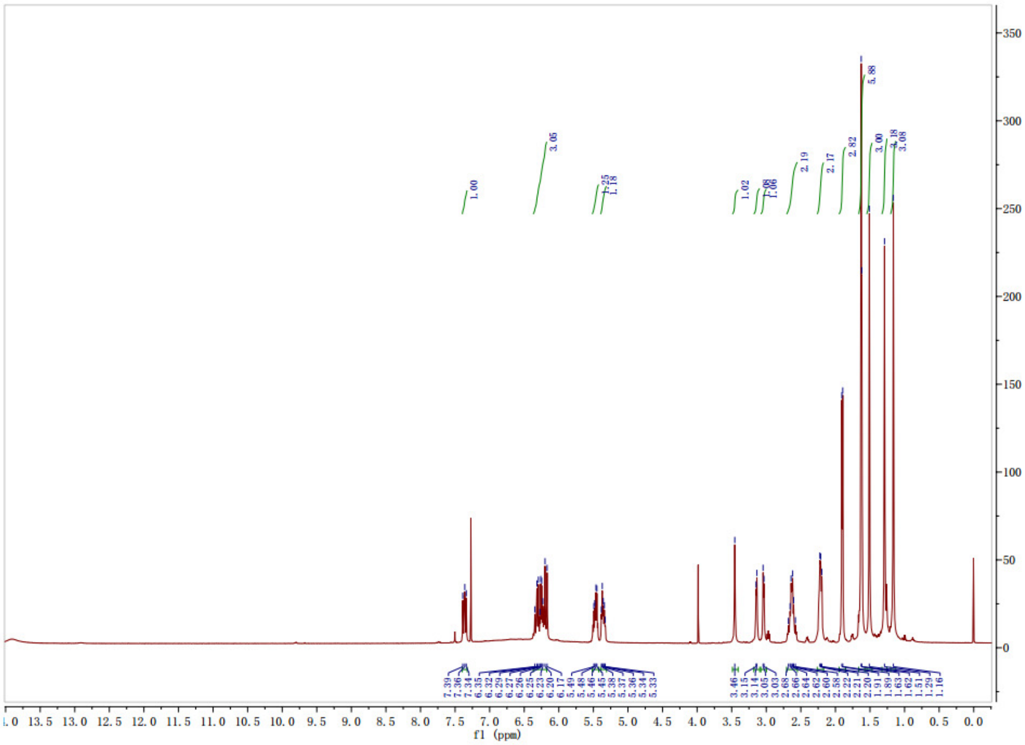

Figure S22. ^{13}C NMR (125 MHz, $CDCl_3$) spectrum of compound **4**.

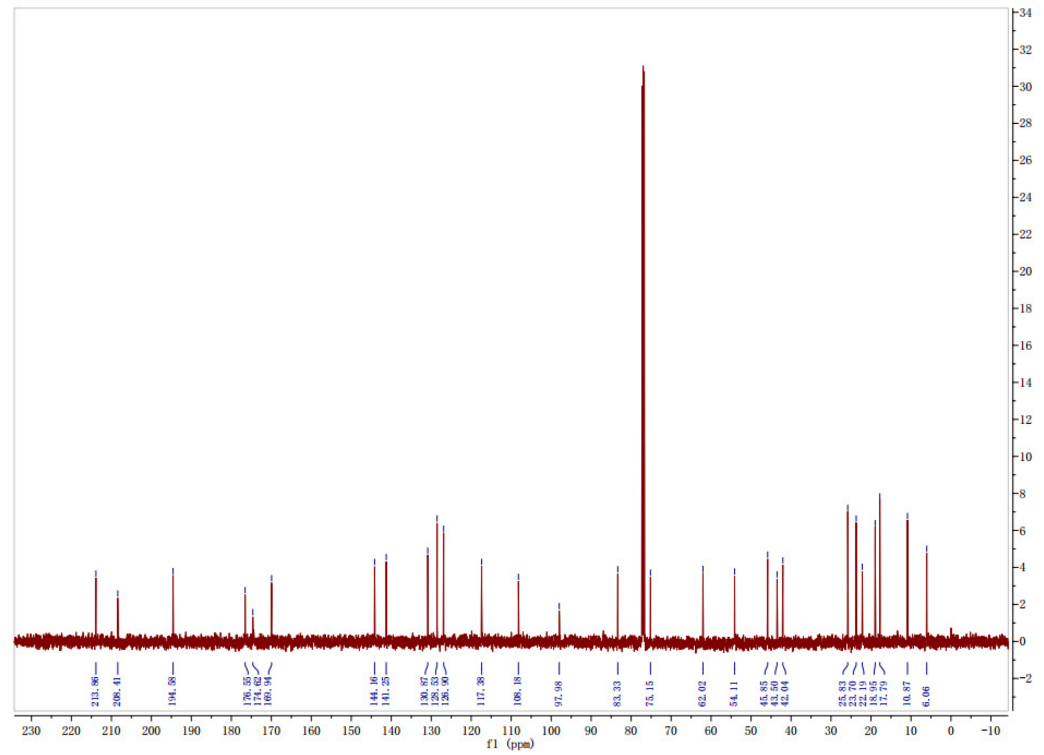

Figure S23. ^{1}H NMR (600 MHz, $CDCl_3$) spectrum of compound **7**.

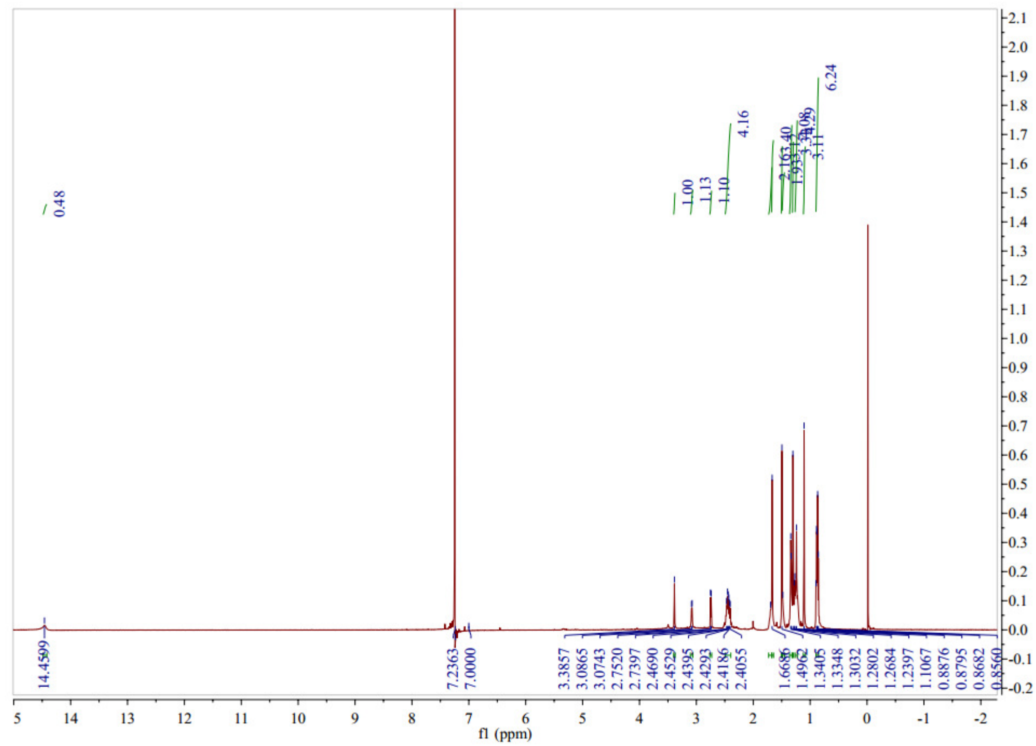

Figure S24. ^{13}C NMR (150 MHz, $CDCl_3$) spectrum of compound **7**.

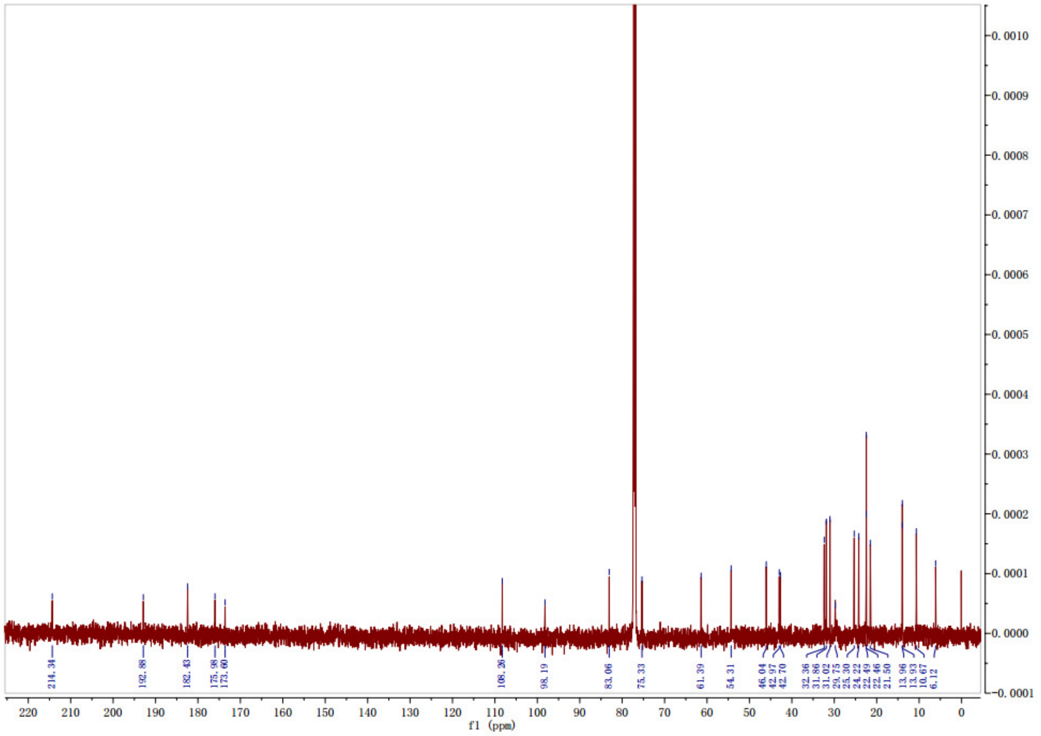

Figure S25. HSQC (600 MHz, $CDCl_3$) spectrum of compound **7**.

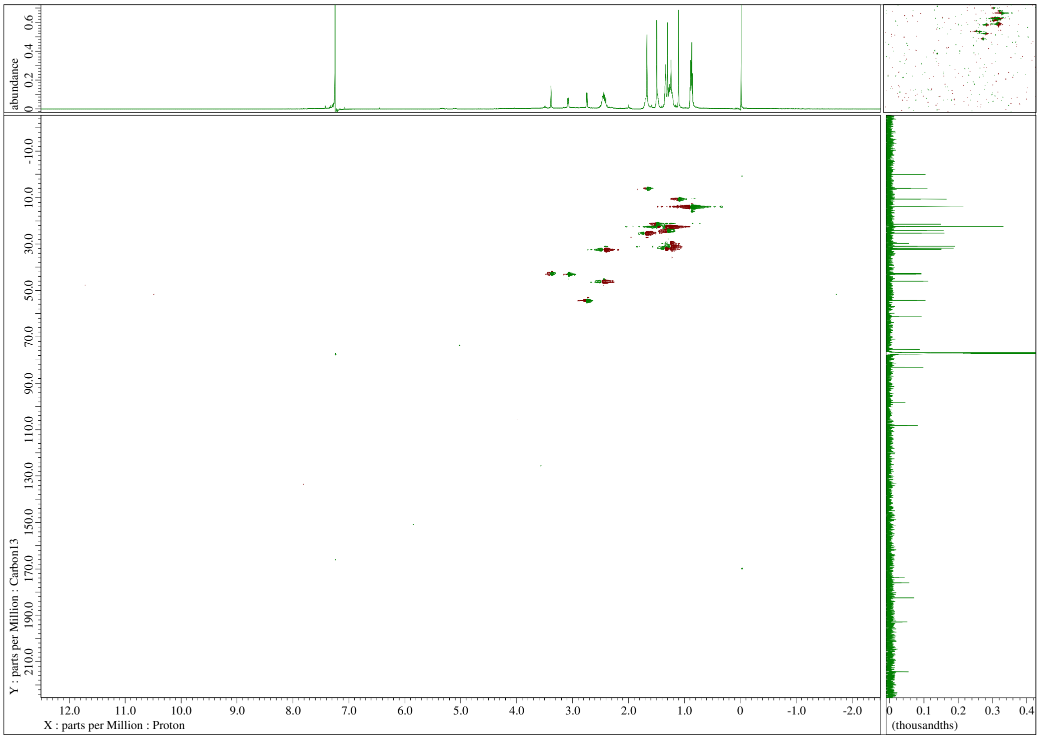

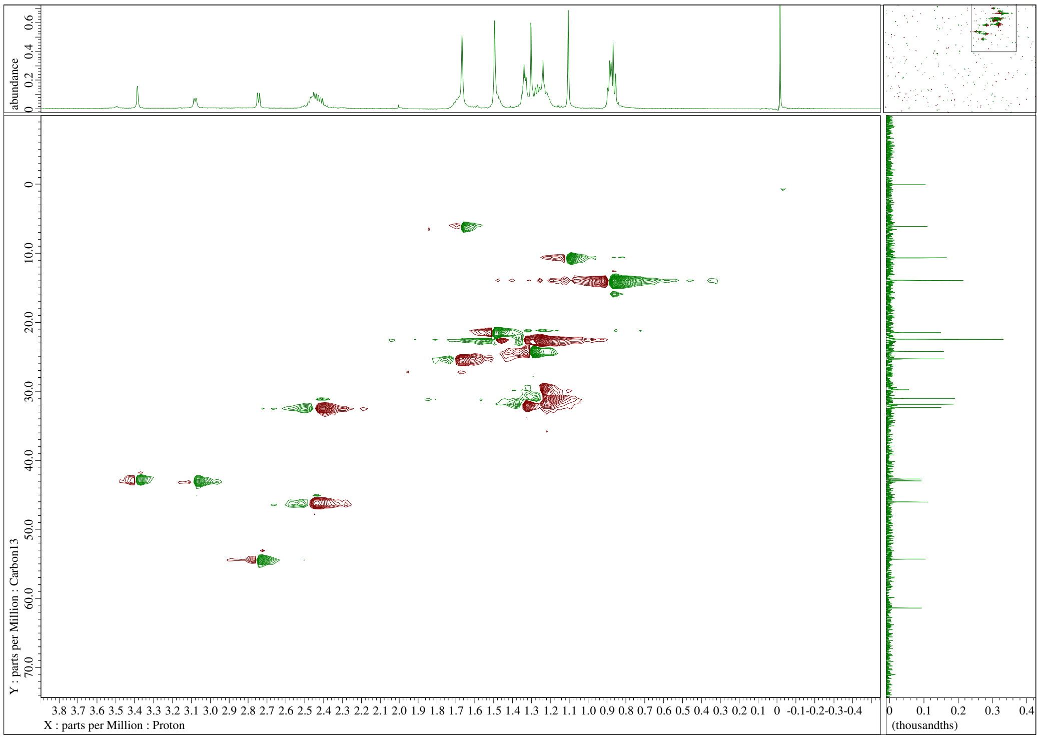

Figure S26. ^{1}H-^{1}H COSY (600 MHz, $CDCl_3$) spectrum of compound **7**.

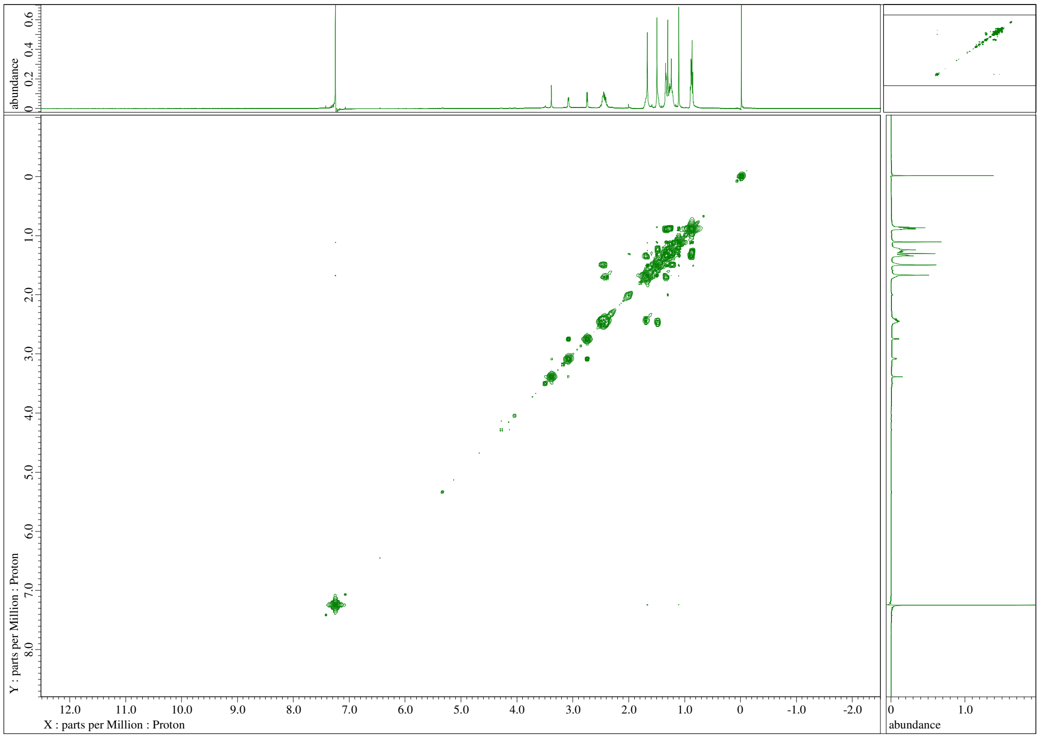

Figure S27. HMBC (600 MHz, $CDCl_3$) spectrum of compound **7**.

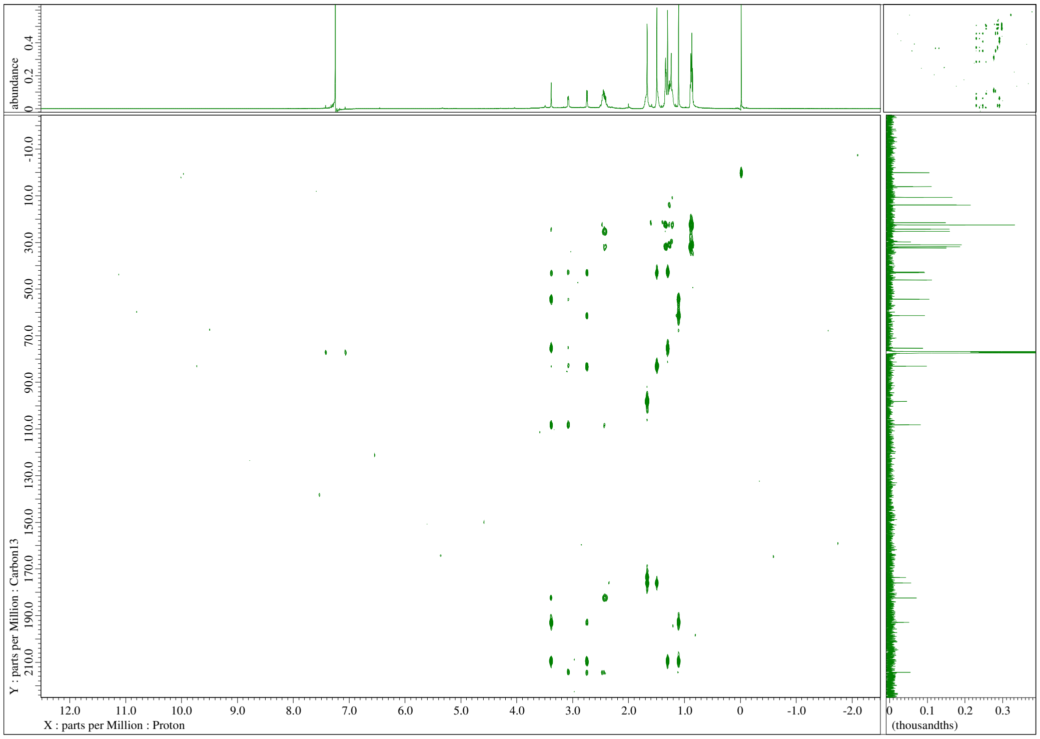

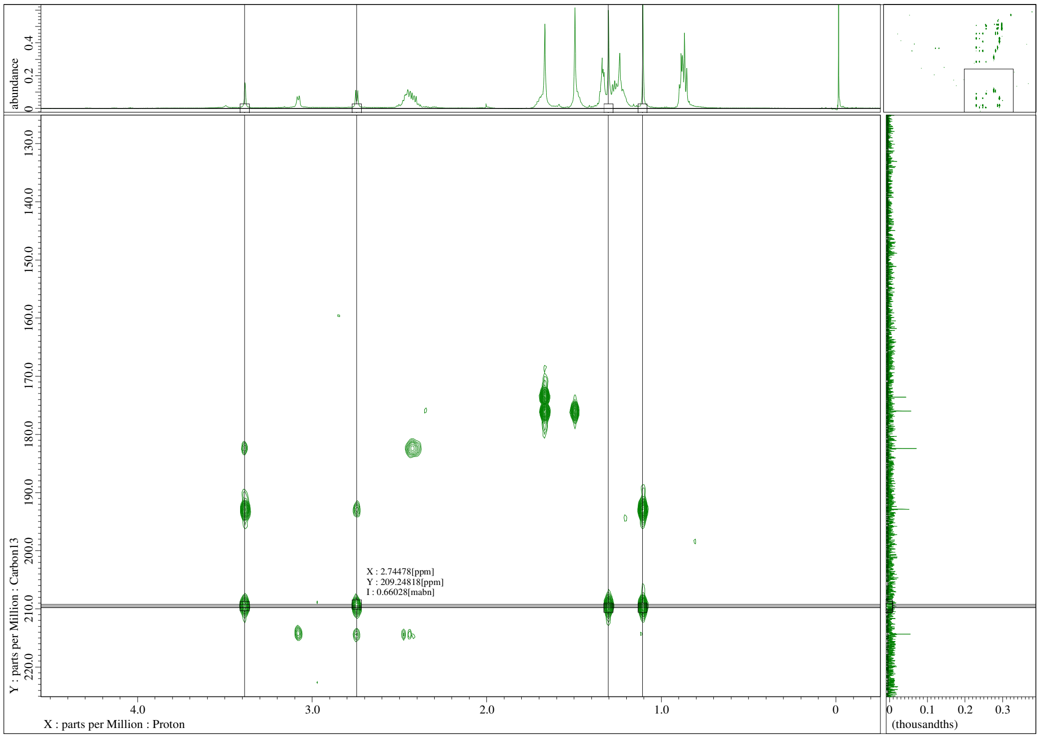

Figure S28. HRESIMS spectrum of compound **7**.

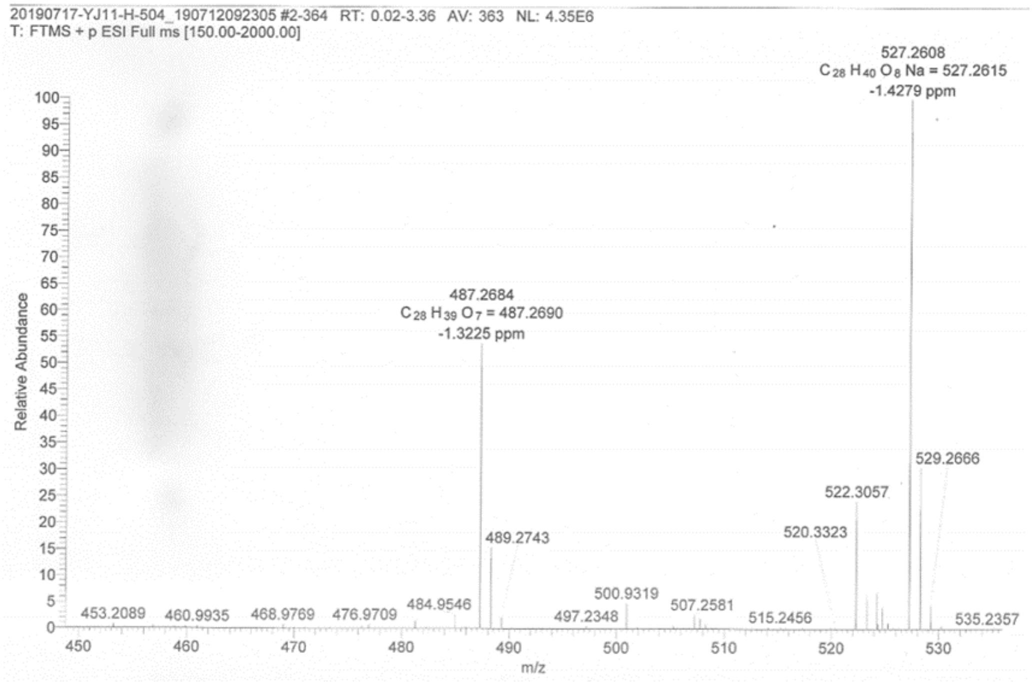

Supplement: Supplementary file 1 [file marinedrugs-17-00446-s001.pdf]
